# Computational elucidation of the reaction mechanism for synthesis of pyrrolidinedione derivatives *via* Nef-type rearrangement – cyclization reaction†

**DOI:** 10.1039/c7ra11908a

**Published:** 2018-01-16

**Authors:** Eleonora D. Ilieva, Galina P. Petrova, Rositca D. Nikolova, Georgi N. Vayssilov

**Affiliations:** Faculty of Chemistry and Pharmacy, University of Sofia 1, James Bourchier Blvd 1126 Sofia Bulgaria gnv@chem.uni-sofia.bg

## Abstract

This paper reports a quantum chemical study of all stages of a one-pot synthesis of pyrrolidinedione derivatives from nitromethane and coumarin, which includes Michael addition, migration of an oxygen atom (Nef-type rearrangement), and cyclization to a pyrrolidine ring. The energy barrier of deprotonated nitromethane addition to coumarin is 21.7 kJ mol^−1^, while the barrier of proton transfer from the methylene to the nitro group in the nitromethyl group is notably higher, 197.8 kJ mol^−1^. The second stage of the reaction, migration of an oxygen atom within the nitromethyl group, occurs with lowest energy barrier, 142.4 kJ mol^−1^, when it is assisted by an additional water molecule. The last stage – cyclization, passes with a very low energy barrier of 11.9 kJ mol^−1^ but the tautomerization of the nitrosohydroxymethyl group to the hydroxy-*N*-hydroxyiminomethyl, necessary for the process, has an energy barrier of 178.4 kJ mol^−1^. Analogous calculations for the same process with the ethyl ester of 3-coumarin-carboxylic acid as substrate show that the relative energies of the intermediates and transition states are by at most 10–16 kJ mol^−1^ more stable than the corresponding structures with coumarin.

## Abstract 2

A quantum chemical study of all stages of pyrrolidinedione derivative synthesis from nitromethane and coumarin, which includes Michael addition, migration of an oxygen atom (Nef-type rearrangement), and cyclization to a pyrrolidine ring is reported.

## Graphical abstract

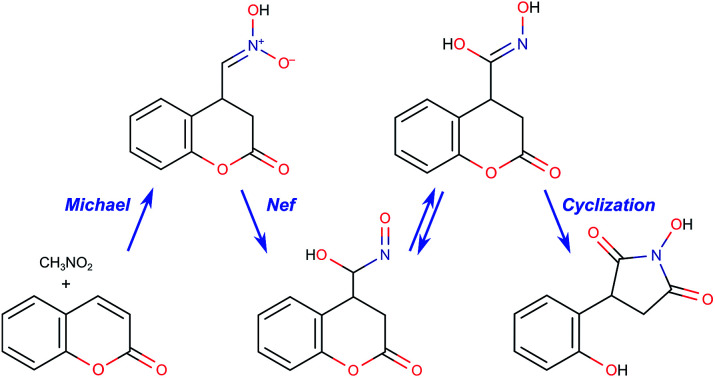

## 1. Introduction

**Scheme 1:**
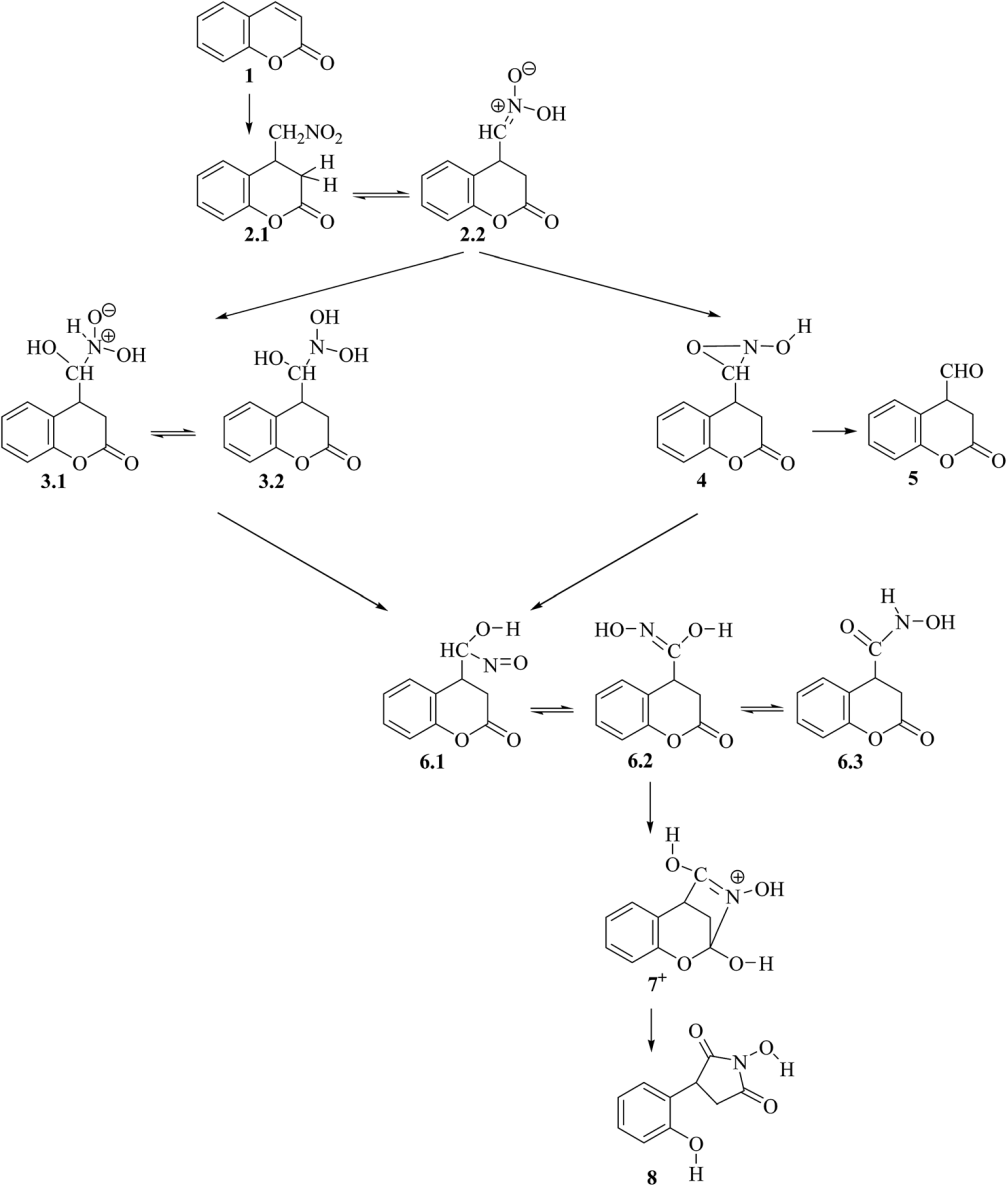


Michael addition followed by a Nef-type rearrangement reaction (Scheme 1) in which coumarin derivatives 1 are transformed into pyrrolidinedione products 8, 1-hydroxy-4-(2-hydroxyphenyl)-2,5-dioxopyrrolidine was reported to occur with an excellent yield.^1,2^ Initially the reaction was reported with 3-phosphonocoumarin, but later we showed that this rearrangement reaction occurs also with other 3-substituted coumarins and the coumarin itself as reactants 2 and can find application in the target synthesis of 3,4-disubstituted pyrrolidine derivatives with potential biological activity.

Numerous compounds with anticonvulsant activity contain five- or six-membered heterocyclic rings, one or two carbonyl groups, as well as an aromatic system,^3,4^ as in the pyrrolidinedione derivatives obtained *via* the reported process. In particular, it was reported that some 3,4-disubstituted pyrrolidinediones are successfully used for the treatment of epilepsy,^5–8^ which is one of the most common neurological disorders affecting approximately 1% of the population worldwide according to the World Health Organization.^9,10^ Despite the progress in understanding the pathogenesis of seizures, the current therapy remains still ineffective or causes serious side effects,^11–13^ which underlines the necessity of new drugs with higher efficiency and less side effects.

In the reports about the synthesis of pyrrolidinedione derivatives from coumarins^1,2^ a reaction scheme for the mechanism of the entire process is proposed based on the experimental observations. It involves Michael addition of nitromethane to the coumarin, followed by Nef rearrangement of the nitromethyl substituent into nitroso-hydroxymethyl group and cyclization into pyrrolidine ring accompanied by the lactone ring opening. In the current study we aim at elucidating the reaction mechanism by means of quantum chemical modelling based on density functional theory (DFT) and Møller–Plesset perturbation theory of second order (MP2) on the example of the coumarin transformation under the reaction conditions. In order to clarify the reaction mechanism, we determined the relative stability of intermediate species and the energy barriers of various possible elementary steps considering also the possible tautomeric structures of the intermediates and their interconversion. We also took into account the actual reaction conditions at each stage of the process *via* modelling the effect of the solvent and presence of basic or acidic reagent in the reaction mixture.

Key step of the proposed mechanism is a rearrangement reaction that coincides with the first step of the Nef reaction for conversion of nitro compounds into carbonyl compounds.^14–16^ Ballini *et al.*^17^ proposed a mechanism of the process, involving a base catalyzed tautomerization of the nitro compound to nitronate salt, and its protonation followed by hydrolysis of the C=N double bond to nitroso intermediate, which further decomposes to carbonyl compound, hyponitrous acid and water. In the reaction of secondary nitroalkanes promoted by DBU (1,8-diazabicyclo[5.4.0]undec-7-ene) under basic homogeneous conditions,^18^ the rearrangement of the protonated intermediate could not be accomplished and thus, a mechanism trough a three member oxaziridine ring was proposed.

Computational studies of the Nef reaction mechanism and the rearrangement are rather rare. Bock *et al.*^19^ reported that the activation energy of the tautomerization process of a single nitromethane molecule is very high, 335 kJ mol^−1^, at HF/6-31G** level. By this reason, *aci*-nitro compounds are obtained by acidification of their salts. Zeman *et al.*^20^ investigated the mechanism of formation of *aci*-nitromethane in presence of water or ammonia using DFT method, B3LYP/6-31G**, and found that the proton transfer in this case has much lower activation barrier, 151 kJ mol^−1^ and 104 kJ mol^−1^, when assisted by water or ammonia molecule, respectively. Khrapkovskii *et al.*^21^ showed that the activation energy for formation of the nitromethane *aci*-forms is 317.8 (319.4) kJ mol^−1^ at MP3/RHF(DZV-1d) (CCD/RHF(DZV-1d)) in good agreement with the results of Bock *et al.*,^19^ and the reaction is endothermic by 69.5 (71.8) kJ mol^−1^. These model studies concern the initial tautomerization of nitromethane but the Nef rearrangement mechanism *via* protonation of the *aci*-nitro intermediate or *via* formation of oxaziridine ring has not been modelled so far by computational methods. By this reason, we modelled the Nef rearrangement in various ways as a component of the entire process of 3-nitromethyl coumarin transformation into pyrrolidinedione derivative.

## 2. Results and discussion

The whole reaction mechanism involves three main stages depending on the type of reactions (for notation of structures and numbering of the atoms see Scheme 1 and Section 4):^1,2^

**Fig. 1:**
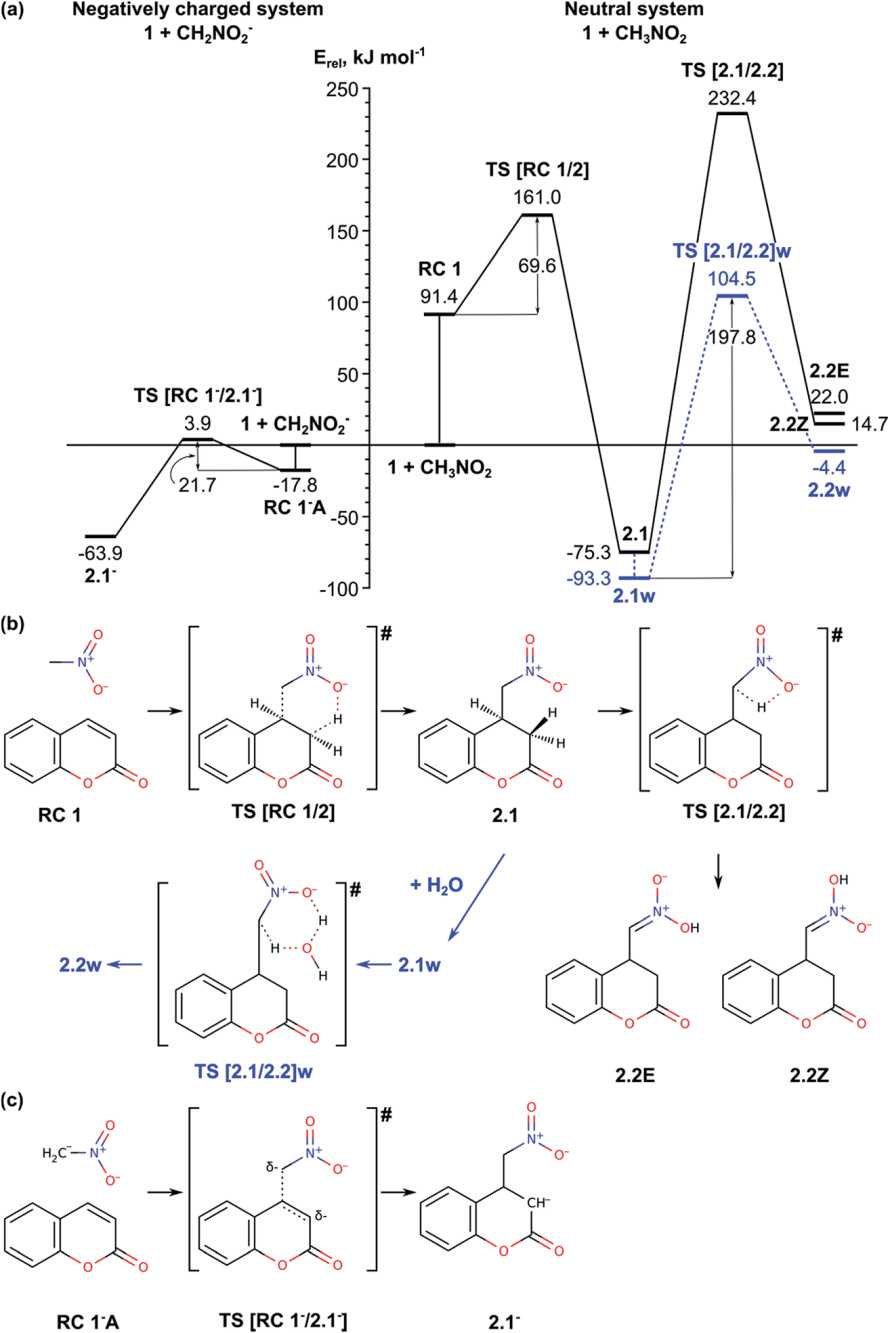
(a) Energy diagram of Michael addition to coumarin 1 of nitromethane, CH_3_NO_2_ (right-hand side), and deprotonated nitromethane, CH_2_NO_2_^−^ (left-hand side). Schematic representation of the reaction paths for CH_3_NO_2_ (b), and CH_2_NO_2_^−^ (c).

**Fig. 2:**
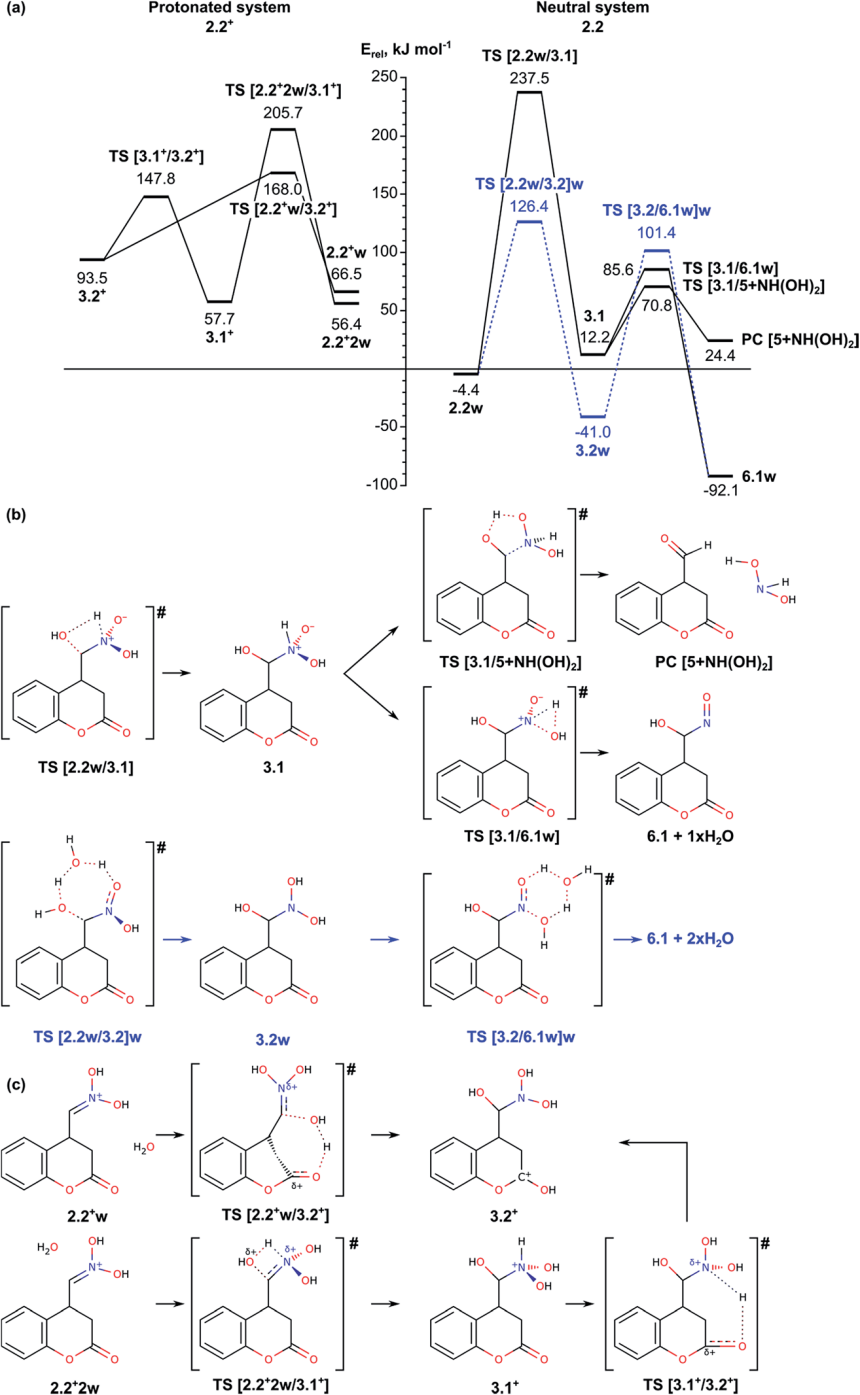
(a) Energy diagram of the mechanism for O-atom migration assisted by a water molecule (right-hand side) and assisted by a water molecule in acidic solution (left-hand side). (b) Schematic representation of the reaction paths for water assisted reaction (b), and water assisted reaction in acidic conditions (c).

**Fig. 3:**
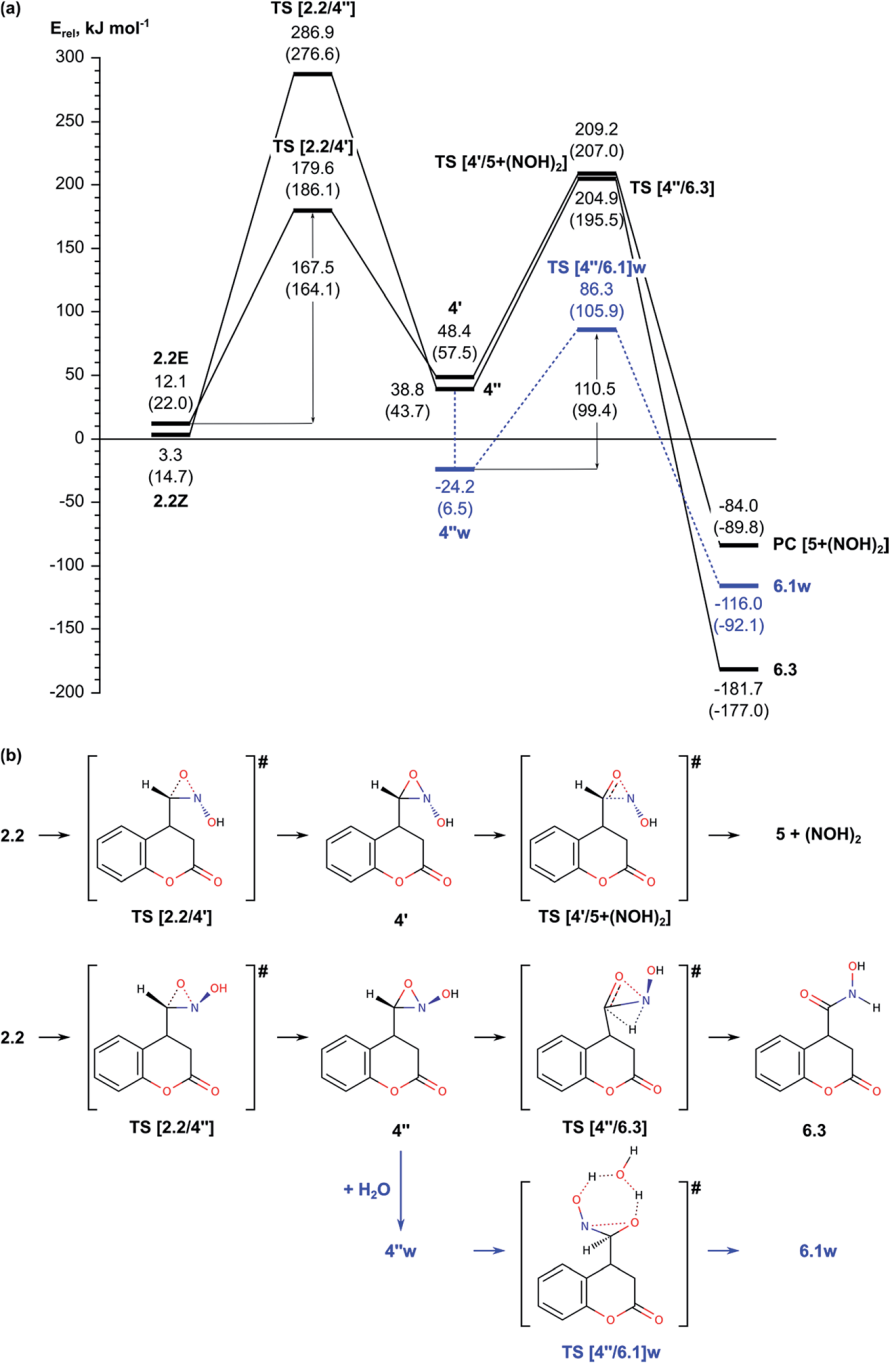
(a) Energy diagram of the mechanism of O-atom migration *via* formation of a three-membered oxaziridine cycle in gas phase; relative energies in solvent are provided in parenthesis for comparison. (b) Schematic representation of the reaction paths.

**Fig. 4:**
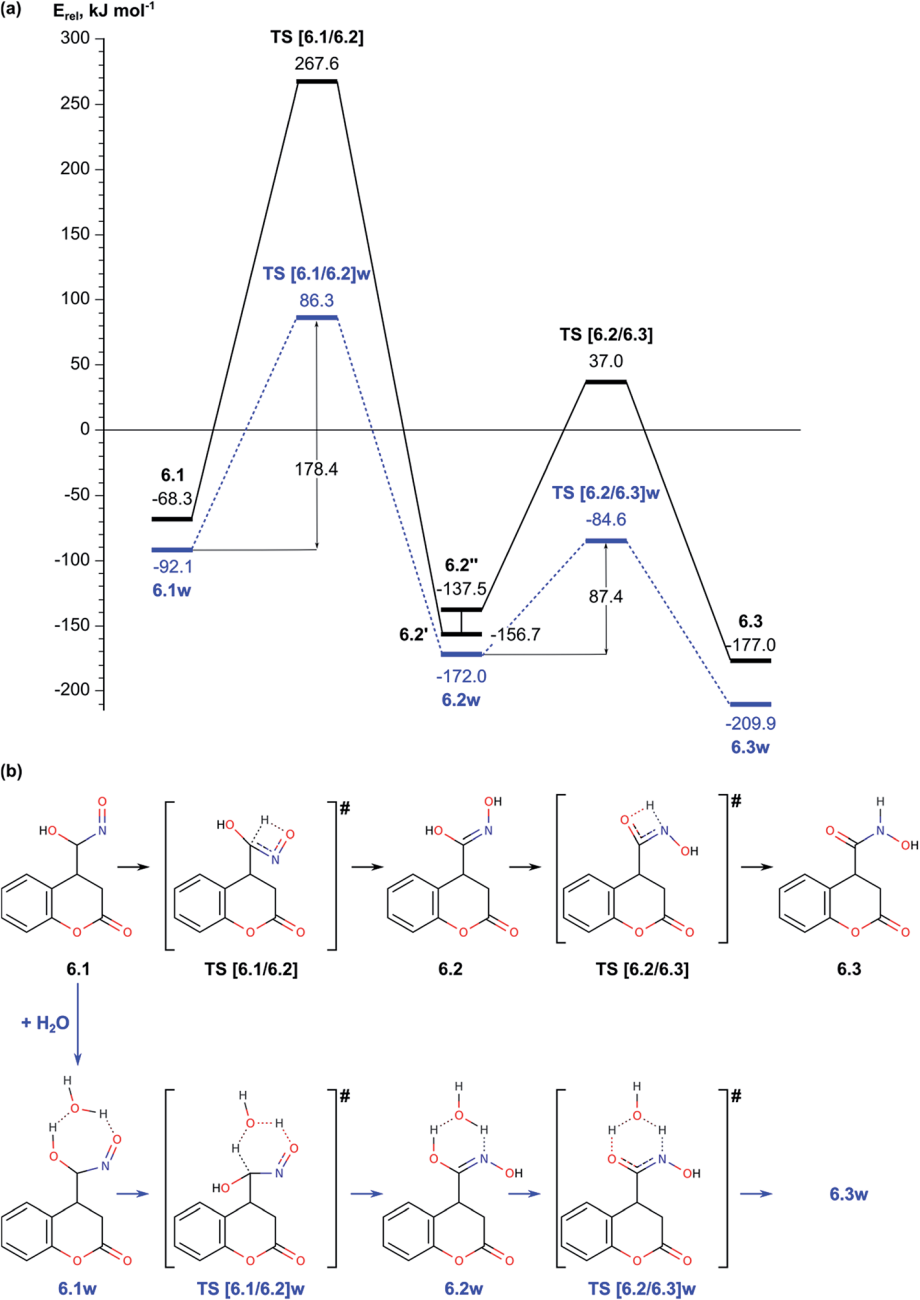
(a) Energy diagram of the transformation between different tautomeric forms of the nitrosohydroxymethyl group in intermediate 6. (b) Schematic representation of the reaction paths.

**Fig. 5:**
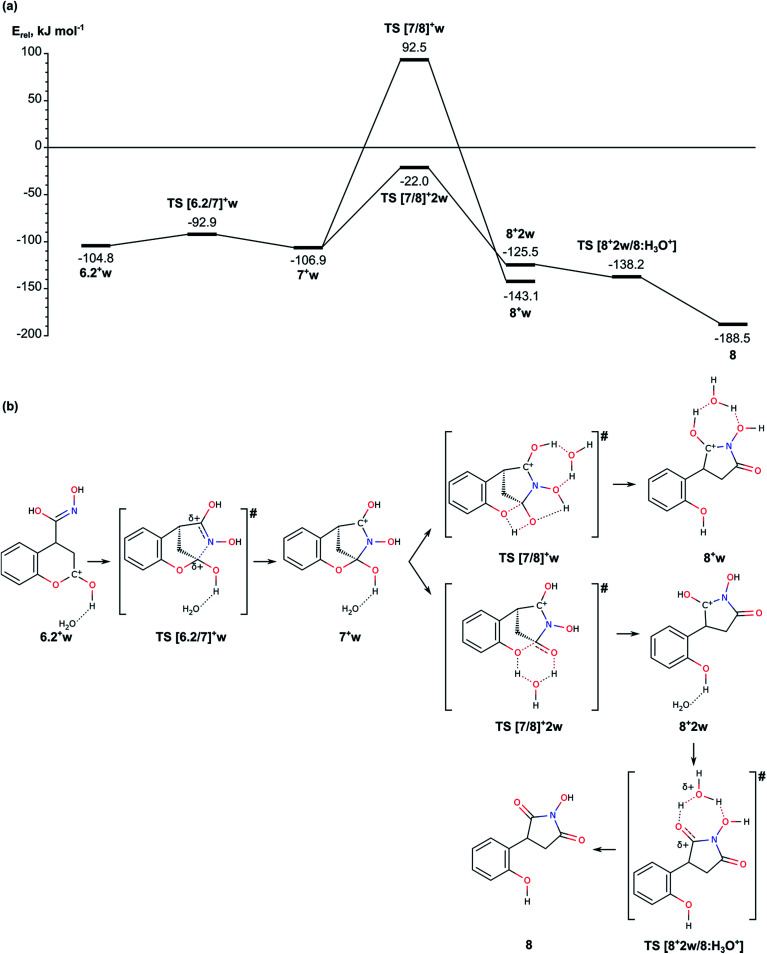
(a) Energy diagram of mechanism for formation of pyrrolidine ring from intermediate 6.2. (b). Schematic representation of the reaction paths.

2.1. Michael addition reaction of CH_3_NO_2_ to the C4 carbon atom of the coumarin (Fig. 1);

2.2. Nef-type rearrangement of the nitromethyl group to nitroso-hydroxymethyl group including tautomerization of the nitromethyl group to *aci*-nitromethyl followed by oxygen migration from N to C atom (Fig. 2–4);

2.3. Tautomerization of the nitroso-hydroxymethyl group, followed by cyclization *via* formation of the N–C2 bond and opening of the lactone ring (Fig. 5).

The optimized structures of all intermediate and transition state (TS) structures are presented on Fig. S1–S6 in the ESI,† including some important interatomic distances. The energy values discussed in the text and reported in the figures are obtained with single point MP2 (Møller–Plesset perturbation theory – second order correction) calculations taking into account the solvent by continuum model, as described in the Computational details section, where also the relevant references are provided. In Table S1 in ESI† we provided the values for the reaction and activation energies of various reaction steps as well as the values of the zero-point energy correction and entropy contribution to the Gibbs free energies. The influence of those corrections on the analysis of the reaction mechanism are discussed in Section 2.4. In our modelling we followed the experimental reaction conditions, described earlier.^1,2^

### 2.1 Michael addition

The first stage of the process, the addition of CH_3_NO_2_ to the coumarin 1 resulting in formation of 4-(nitromethyl)-2-oxochroman 2, is favourable by 75.3 kJ mol^−1^ (Fig. 1, Table S1†). First, we modelled the interaction of coumarin 1 with the *aci*-form of nitromethane CH_2_NO_2_H, which is by 104 kJ mol^−1^ less stable than nitromethane. The relative energy of the formed reaction complex RC 1 is 91 kJ mol^−1^ and it is more stable than the separated system [1 + CH_2_NO_2_H] by only 13 kJ mol^−1^. TS [RC 1/2] is concerted TS with relative energy 161 kJ mol^−1^, in which the formation of C–C bond and the proton transfer from –NO_2_H to C3 atom of the coumarin take place simultaneously. Considering the rather high relative energies of RC 1 and TS [RC 1/2], we also modelled the reaction with initially deprotonated nitromethane. Deprotonation of nitromethane (p*K*_a_ ∼ 10.2) can occur under experimental conditions due to the presence of triethylamine (p*K*_a_ ∼ 10.7).^1,2^ We found two stable complexes of CH_2_NO_2_^−^ with the coumarin: RC 1^−^A and RC 1^−^B with the same relative energies, −18 kJ mol^−1^. RC 1^−^B is formed as a result of weak H-bonding between the oxygen atoms from the deprotonated nitromethane and aromatic hydrogens of the coumarin as the H-bond lengths are about 2.0 Å. The other reactant complex, RC 1^−^A, has a structure closer to the following TS with nitromethane located at distance 2.6 Å above C4. The activation energy for formation of the anionic intermediate 2.1^−^ from RC 1^−^A*via*TS [RC 1^−^/2.1^−^] is only 21.7 kJ mol^−1^. The final product of the Michael addition reaction can be obtained by a direct protonation of C3 atom in intermediate 2.1^−^ by Et_3_NH^+^, formed at the initial deprotonation of the nitromethane.

The tautomerization of intermediate 2.1 to 2.2 is modelled for the neutral system (Fig. 1, right hand side of panel (a); panel (b)). The proton transfer from the methylene to the nitro group through TS [2.1/2.2] is accomplished by forming a four membered ring with proton transfer angle estimated to 104.1° and the TS structure has high relative energy, 232.4 kJ mol^−1^. However, in solvent (water in the experiment) the process can be assisted by a water molecule. Thus, the proton transfer can be accomplished *via* a hexagonal transition state TS [2.1/2.2]w with *E*_rel_ = 104.5 kJ mol^−1^ and activation barrier of 197.8 kJ mol^−1^, *i.e.* slightly higher (by *ca.* 50 kJ mol^−1^) than the value reported earlier for the same process in nitromethane assisted by water at B3LYP/6-311++G(d, p), 151.5 kJ mol^−1^.^20^ In the hexagonal TS the proton transfer angles are partially relaxed, 143.4 and 152.7°. The TS is found late with respect to the proton transfer from the methylene group to the water molecule but early with respect to the transfer to the nitro group. Both TS structures result in the *Z*-isomer of 2.2. The relative energy of 2.2Z without explicit water is estimated to 14.7 kJ mol^−1^ (the corresponding *E*-isomer 2.2E is less stable by 7.3 kJ mol^−1^), while the complex with water is found slightly more stable, by 10.3 kJ mol^−1^. In both cases, however, the tautomerization of 2.1 to 2.2 remains highly endothermic process requiring more than 60 kJ mol^−1^ (Table S1†).

In summary, the Michael reaction of coumarin 1 with deprotonated nitromethane passes through activation barrier of 21.7 kJ mol^−1^. The tautomerization of structure 2.1 to 2.2 can be accomplished with an additional proton-donor/proton-acceptor molecule and the energy barrier of this step is still high, 197.8 kJ mol^−1^.

### 2.2 Oxygen migration (Nef rearrangement)

The next step in our study includes modelling of the mechanisms for oxygen migration from the NO_2_H group to the CH_2_ group (Nef rearrangement). We considered both the ideas already proposed in the literature^14–16^ and some alternative reaction paths, not discussed so far. In order to account properly for the reaction conditions, we modelled four possible mechanisms depending on the solvent and presence of acidic/basic compounds: (i) reaction in acidic aqueous media, assisted by a water molecule; (ii) reaction through formation of a three-membered oxaziridine ring; and (iii) reaction assisted by triethylamine. Since the mechanism of the Nef rearrangement assisted by triethylamine includes transition states with rather high activation energy, they are described shortly in the ESI (see Fig. S4 and S7†).

#### (i) Reaction in acidic aqueous media, assisted by a water molecule

O-Atom migration from the *aci*-nitro group to the neighboring carbon atom was modelled in the isolated neutral molecule as well as a process assisted by one or two H_2_O molecules or with a protonated nitro group also assisted by a H_2_O molecule. The obtained results and schematic representation of the reaction paths are presented on Fig. 2, while the structures of the optimized intermediate and TS structures are shown on Fig. S2.† In presence of water actual oxygen transfer occurs by subsequent addition of water to the methylene C atom of intermediate 2.2 and elimination of water from the *aci*-nitro group leading to the nitroso-hydroxymethyl intermediate 6.1. The water-addition step is modelled in two ways through TS [2.2w/3.1] and TS [2.2w/3.2]w leading to formation of intermediates 3.1 (azinic acid type) and 3.2 (*N*,*N*-dihydroxyamino type), respectively. Both TS structures include formation of O–C bond between the water molecule and the methylene C atom. If proton transfer from the same H_2_O molecule to the N atom of the NO_2_H group occurs simultaneously with the formation of the O–C bond, then the reaction barrier *via* the tetragonal TS [2.2w/3.1] for formation of intermediate 3.1 is rather high, 241.9 kJ mol^−1^. If the process is assisted by additional water molecule, *via* the so-called proton shuttle mechanism,^22–24^ then the proton transfer is accomplished directly to negatively charged oxygen center in the *aci*-nitro group, which is much better proton acceptor than the nitrogen center. By this reason, the reaction barrier *via* the heptagonal TS [2.2w/3.2]w for the process assisted by a second water molecule is 130.8 kJ mol^−1^, much lower than the previous one. On the other hand, the intermediate complex 3.2w (formed between intermediate 3.2 and the remaining water molecule) has negative relative energy, −41.0 kJ mol^−1^, *i.e.* it is more stable than both intermediates 2.2w and 3.1.

As can be seen from Fig. 2a (right-hand side), intermediate 3.1 can be transformed through low energy barriers *via*TS [3.1/5 + NH(OH)_2_] with *E*_act_ = 58.6 kJ mol^−1^ or TS [3.1/6.1]w, *E*_act_ = 73.4 kJ mol^−1^, either to the Nef reaction product 5 (with elimination of NH(OH)_2_), or to intermediate 6.1 leading subsequently to formation of the pyrrolidine ring. The formation of the product complex in the first case is endothermic most probably due to low stability of the side product dihydroxyamine. In the second case the reaction proceeds through a trigonal TS structure for proton transfer from the NH group to the hydroxyl to release a water molecule. Transition to intermediate 6.1 was modelled also from intermediate 3.2w, *i.e.* involving a water molecule to facilitate the proton transfer in 3.2 from one of the *N*-hydroxyls to the other and releasing two H_2_O molecules. The energy barrier for the transition state TS [3.2/6.1w]w, 142.4 kJ mol^−1^, is almost twice as high as the barrier through TS [3.1/6.1]w both due to destabilization of the transition state and the higher stability of the initial intermediate 3.2w.

Since the reaction takes place in acidic solution, we also modelled the addition/elimination of water when the substrate is initially protonated 2.2^+^w (Fig. 2a, left-hand side). The protonation of the nitronic group in intermediate 2.2 from H_3_O^+^ may result in the formation of the complexes 2.2^+^w and 2.2^+^2w, which are energetically disfavored by 70.9 and 60.8 kJ mol^−1^ with respect to 2.2. The addition of a water molecule to C=N bond in 2.2^+^2w including simultaneous formation of C–O and N–H bonds *via* transition state TS [2.2^+^2w/3.1^+^] leads to 3.1^+^, a protonated form of 3.1. The energy barrier of this step (above 150 kJ mol^−1^) is rather high, similarly to the corresponding non-protonated transition state TS [2.2w/3.1] (*E*_act_ = 241.9 kJ mol^−1^). Alternatively, the proton from the water molecule can be transferred to the carbonyl oxygen of the lactone forming protonated form of 3.2, 3.2^+^. The energy barrier for this process is notably lower, 101.5 kJ mol^−1^. Once formed, intermediate 3.2^+^ can be deprotonated to 3.2, which can be transformed into 6.1 as it is described above.

In summary, the transformation of the nitromethyl intermediate 2 to the nitroso-hydroxymethyl intermediate 6.1 can occur *via* initial H transfer and addition/elimination of water with assistance of a second water molecule. The important transition states along this part of the reaction path are TS [2.1/2.2]w (tautomerization TS, see Section 2.1), TS [2.2w/3.2]w and TS [3.2/6.1w]w with relative energies 104.5, 126.4 and 101.4 kJ mol^−1^ and *E*_act_ values of 197.8, 130.8 and 142.4, respectively. Alternatively, aldehyde can be obtained as a Nef reaction product *via* the intermediates 3.1.

#### (ii) Reaction through formation of a three-membered oxaziridine ring

Since the Nef reaction occurs in the absence of water, we modelled direct transfer of oxygen atom from N to C *via* formation of a three-membered oxaziridine ring, as suggested previously.^18^ In this case the discussion is based on values for the relative energy obtained in gas phase (the values in implicitly considered water solvent are provided in parentheses). The corresponding three-center transition states TS [2.2/4′] or TS [2.2/4′′] are presented on Fig. 3b as schematic representations and on Fig. S3† as optimized structures. The difference in the relative energies of the two TS structures is estimated to 107.3 (90.5) kJ mol^−1^ as the activation energy through TS [2.2/4′], 167.5 (164.1) kJ mol^−1^, is lower.

This step of the reaction mechanism results in the formation intermediate 4 with C–O–N oxaziridine ring with two conformations depending on the position of the hydroxyl group with respect to the oxaziridine plain, 4′ and 4′′, bellow or above, respectively. The conformers 4′ and 4′′ are by 45.1 (42.7) and 35.5 (29.0) kJ mol^−1^, respectively, less stable than the previous intermediate along this reaction path, 2.2Z. The attempt to achieve complete O-migration to the C atom in 4′ resulted in C–N bond cleavage, accompanied by formation of aldehyde group (attached at C4 atom of the coumarin) and NOH moiety, which essentially are the Nef reaction products.^14^ The energy barrier of this step through TS [4′/5 + (NOH)_2_] is relatively high, 160.8 (149.5) kJ mol^−1^ with respect to 4′, but the final product of the Nef reaction is very stable, with *E*_rel_ = −84.0 (−89.8) kJ mol^−1^. Due to the high barrier for formation of oxaziridine intermediate 4, the Nef reaction typically occurs at high temperature.

In summary, the results described in the Subsection 2.2 suggest that the migration of oxygen atom (Nef rearrangement) proceeds most easily when it is assisted by a water molecule in acidic media *via* transition states TS [2.2w/3.2]w and TS [3.2/6.1w]w with relative energies 126.4 and 101.4 kJ mol^−1^ and energy barriers of 130.8 and 142.4 kJ mol^−1^, respectively. In absence of water the energetically most favored reaction path includes formation of three-centered oxaziridine ring with energy barrier of 164.1 kJ mol^−1^ (TS [2.2/4′]) and its decomposition with *E*_act_ = 151.8 kJ mol^−1^ (TS [4′′/6.3]). The other modelled mechanism, assisted by triethylamine, has higher energy barrier and thus, is unlikely.

### 2.3 Cyclization

Before we started the modelling of cyclization leading to formation of pyrrolidine ring, we studied the tautomeric equilibrium within the nitrosohydroxymethyl group in intermediate 6.1 (Fig. 4) in order to identify the most stable tautomer and the tautomer with the most suitable structure for nucleophilic attack to the carbonyl C2 center in the lactone ring. The modelled tautomeric forms are hydroxy-*N*-hydroxyiminomethyl 6.2 and hydroxyamide 6.3. Both forms are found more stable than 6.1 by 88.4 and 108.7 kJ mol^−1^, respectively. The conversions between different tautomeric forms assisted by an additional water molecule, acting as a proton shuttle, occur *via* transitions states TS [6.1/6.2]w and TS [6.2/6.3]w with energy barriers of 178.4 and 87.4 kJ mol^−1^, respectively. Although tautomer 6.3 (*N*-hydroxycarboxamide) is the most stable, its structure is not suitable for further cyclization since the lone electron pair of the nitrogen atom does not have an appropriate orientation. By this reason we modelled the cyclization using tautomer 6.2 (Fig. 4 and 5).

In order to facilitate the formation of the N–C2 bond we protonated the carbonyl group of the lactone ring in 6.2 since the reaction takes place in the acidic aqueous solution. The obtained structure 6.2^+^w is more stable than 6.2 and 6.2w by 51.9 and 67.2 kJ mol^−1^, respectively. The formation of pyrrolidine ring from the protonated species 6.2^+^w occurs very easy *via* a transition state TS [6.2/7]^+^w with energy barrier of only 11.9 kJ mol^−1^.

The next stage of the process, the lactone ring opening, is a synchronous process which includes a proton transfer between two oxygen atoms – from the protonated carbonyl group to the lactone oxygen atom. If the proton transfer occurs as a direct transfer *via* a tetragonal transition state TS [7/8]^+^w, the energy barrier is 199.4 kJ mol^−1^, which is reduced to 84.9 kJ mol^−1^, when the transfer is assisted by a water molecule *via* transition state TS [7/8]^+^2w with relative energy −22.0 kJ mol^−1^.

The last step of the process – the deprotonation of [C=OH]^+^ group at the pyrrolidine and formation of the product 8, is accomplished spontaneously as the structure corresponding to a transition state pseudo TS [8^+^2w/8:H_3_O^+^] with relative energy −138.2 kJ mol^−1^ (Fig. 5) is slightly more stable than the intermediate preceding it.

In summary for the stage of cyclization and formation of the pyrrolidine ring occurs *via* transition state with low energy barrier, only 11.9 kJ mol^−1^, when the carbonyl lactone group is preliminarily protonated. The tautomerization of structure 6.1 to 6.2, which is the structure suitable for accomplishment of the cyclization step, has relatively high barrier of 178.4 kJ mol^−1^. The ring opening reaction of the lactone ring requires activation energy of 84.9 kJ mol^−1^.

### 2.4 Corrections to the electronic energies

The analysis in this section is based on the influence of some correction to the reaction and activation electronic energies of various reaction steps. Those corrections include the zero-point energy correction and entropy contribution to the Gibbs free energies at 298.15 K (Table S1 in ESI†). In most cases the influence of those contributions does not affect substantially the conclusions based on the electronic energy values, discussed above, the differences are within 10 kJ mol^−1^.

In the first stage, Michael addition, the additional energy contributions increase the activation energy for TS [RC 1/2] by 20.1 kJ mol^−1^ and decrease the barrier for TS [2.1/2.2] by 13.7 kJ mol^−1^. The barrier for the processes in the negatively charged system is also increased by 16.4 kJ mol^−1^.

The effect of the additional corrections are the most substantial for the activation energies for the oxygen migration stage – the energy barrier TS [2.2w/3.2]w increases by 80.5 kJ mol^−1^ and reaches 211.3 kJ mol^−1^, while TS[3.1/6.1w] decreases by 13.4 kJ mol^−1^. The barriers for the protonated system TS [2.2^+^2w/3.1^+^] and TS [2.2^+^w/3.2^+^] increase by 17.3 and 19.6 kJ mol^−1^, respectively.

The energy barriers for Nef reaction in neutral system are essentially not affected by the additional corrections, while the barriers TS [4^−^:bH^+^/6.1:b] and TS [4^−^:bH^+^/6.2^−^:bH^+^] for the base-assisted Nef reaction decrease by 33.7 and 35.2 kJ mol^−1^, respectively.

The barriers for the tautomerization and cyclization are affected by at most 15 kJ mol^−1^ for TS [6.2/7]^+^w.

### 2.5 Reaction mechanism with ethyl ester of 3-coumarin-carboxylic acid

Some steps of the reaction mechanism are modelled with the ethyl ester of 3-coumarin-carboxylic acid applying the same computational approach. The obtained results are provided in Table S2 in ESI.† The calculated relative energy of the Michael product 2.1 is −88.7 kJ mol^−1^, *i.e.* it is by 13.4 kJ mol^−1^ more stable than the product from coumarin. In most of the other modelled steps the calculated relative energies of the intermediates and transition states are by 10–16 kJ mol^−1^ more stable than the corresponding structures with coumarin, including the final product which has relative energy of −199.4 kJ mol^−1^. Due to this total stabilization of all species along the reaction path the activation energies of the transition states are hardly changed.

## 3. Conclusions

We have modelled all stages of a rearrangement reaction, reported recently, which includes Michael addition reaction of CH_3_NO_2_ to coumarin, migration of an oxygen atom (Nef-type process) and cyclization to pyrrolidine ring. Reaction media is taken into account as implicit solvent (water) or as species assisting the reaction with explicit H_2_O (1 or 2) or Et_3_N molecule.

The energy barrier of the reaction of addition of deprotonated nitromethane to coumarin 1 is 21.7 kJ mol^−1^ (38.2 kJ mol^−1^ with corrections to the energy), while the barrier of tautomerization of structure 2.1 to 2.2 is notably higher, 197.8 kJ mol^−1^ (196.3 kJ mol^−1^ after corrections). However, the latter process may occur also by subsequent deprotonation/protonation of the molecule but not *via* intramolecular proton transfer. The second stage of the reaction, migration of an oxygen atom within the nitromethyl group, occurs most easily when assisted by additional water molecule. The energy barriers of addition of water and the following dehydration of the intermediate are 130.8 and 142.4 kJ mol^−1^ (211.3 and 163.4 kJ mol^−1^, respectively, after the corrections). The last stage – cyclization, passes with very low energy barrier of 11.9 kJ mol^−1^ but the tautomerization of the intermediate 6.1 to 6.2, that is accomplished before the cyclization, has an energy barrier of 178.4 kJ mol^−1^ (179.5 kJ mol^−1^ after energy corrections).

Some of the highest energy barriers in the complete reaction mechanism correspond to proton transfer reaction steps. Those steps can proceed, however, much faster than the rate, corresponding to the calculated energy barriers due to proton tunnelling, as shown for other organic processes.^25–27^

The analogous calculations for the same process with ethyl ester of 3-coumarin-carboxylic acid as substrate show that the relative electronic energies of the intermediates and transition states are by at most 10–16 kJ mol^−1^ more stable than the corresponding structures with coumarin. This stabilization of the intermediates and the product are likely the reason for the high reaction yields obtained experimentally with the ethyl ester of 3-coumarin-carboxylic acid compared to the yields with coumarin.

## 4. Computational details

All *ab initio* molecular orbital calculations are performed with the Gaussian 09 program suite.^28^ Analytical gradient optimization methods are used to locate the minima, corresponding to reactants, intermediates and products, and saddle points corresponding to transition states. Transition states are identified by finding only one negative eigenvalue of the analytic force constant matrix and by geometric analysis of its eigenvector components. All lowest points of reaction paths are optimized at B3LYP/6-311+G* level^29–34^ and the electronic energies of the obtained structures are refined by single-point calculations at MP2 level^35,36^ with the same basis set. The influence of the solvent (water if else not specified) on the relative energies of the reactants, intermediates and transition states is estimated *via* single point calculations at MP2/6-311+G*:PCM//B3LYP/6-311+G level with the polarized continuum model (PCM) of the solvent.^37–39^ All relative energies (*E*_rel_ in kJ mol^−1^) are calculated with respect to the isolated coumarin and nitromethane molecules and other species when they are present (as water or base molecule). Except for the relative energies of the species, the activation energies (*E*_act_ in kJ mol^−1^) are discussed as the activation energy is estimated with respect to the initial intermediate at each step of the reaction mechanism. All energy values are reported in ESI.†

The zero-point energy correction to the electronic energy and the entropy contribution to the Gibbs free energies for different reaction and activation energies are reported in Table S1 in ESI.† The values are obtained from the frequency calculations. The entropy contribution is calculated at 298.15 K including all types degrees of freedom; the values for the entropy contribution to specific reaction or activation energies taking into account only vibrational degrees of freedom differ by at most 1.3 kJ mol^−1^ from the reported values.

In the discussion of the reaction mechanism the following notation of the different species is used:

– RC and P denote initial reaction complexes and products, respectively;

– Intermediates in different reaction steps are denoted with consecutive Arabic numbers in bold, *e.g.*5, as different tautomers are denoted by an additional digit, *e.g.*5.1, 5.2, *etc.*;

– When the reaction path under consideration involves charged species, the charge is also denoted, *e.g.*3^−^;

– When the reaction path under consideration involves additional proton, water or base molecule, these features are also denoted in the notation by additional letter H, w or b, respectively, *e.g.*3H^+^ or 5.1w;

– When two different orientations of the interacting species are considered, in addition to the general notation described above, the different orientations are denoted by consecutive capital Latin letter at the end of the notation, *e.g.*RC 1^−^A and RC 1^−^B; – TS [m/n] denotes the transition state between intermediates m and n.

## Conflicts of interest

There are no conflicts to declare.

## Supplementary Material

RA-008-C7RA11908A-s001
