# Supplementary material for: Computational elucidation of the reaction mechanism for synthesis of pyrrolidinedione derivatives via Nef-type rearrangement – cyclization reaction
Source: RSC Adv. 2018 Jan 16;8(6):3178–88. doi: 10.1039/c7ra11908a (PMC9077586; doi:10.1039/c7ra11908a)
Supplement: RA-008-C7RA11908A-s001 [file RA-008-C7RA11908A-s001.pdf]

This journal is © The Royal Society of Chemistry 2018

Electronic supplementary information

Computational Elucidation of the Reaction Mechanism for Synthesis of Pyrrolidinedione Derivatives via Nef-type Rearrangement – Cyclization Reaction

Eleonora D. Ilieva, Galina P. Petrova, Rositca D. Nikolova, and Georgi N. Vayssilov*

* Faculty of Chemistry and Pharmacy, Sofia University, 1, James Bourchier Blvd, 1164 Sofia, Bulgaria, E-mail: gnv@chem.uni-sofia.bg

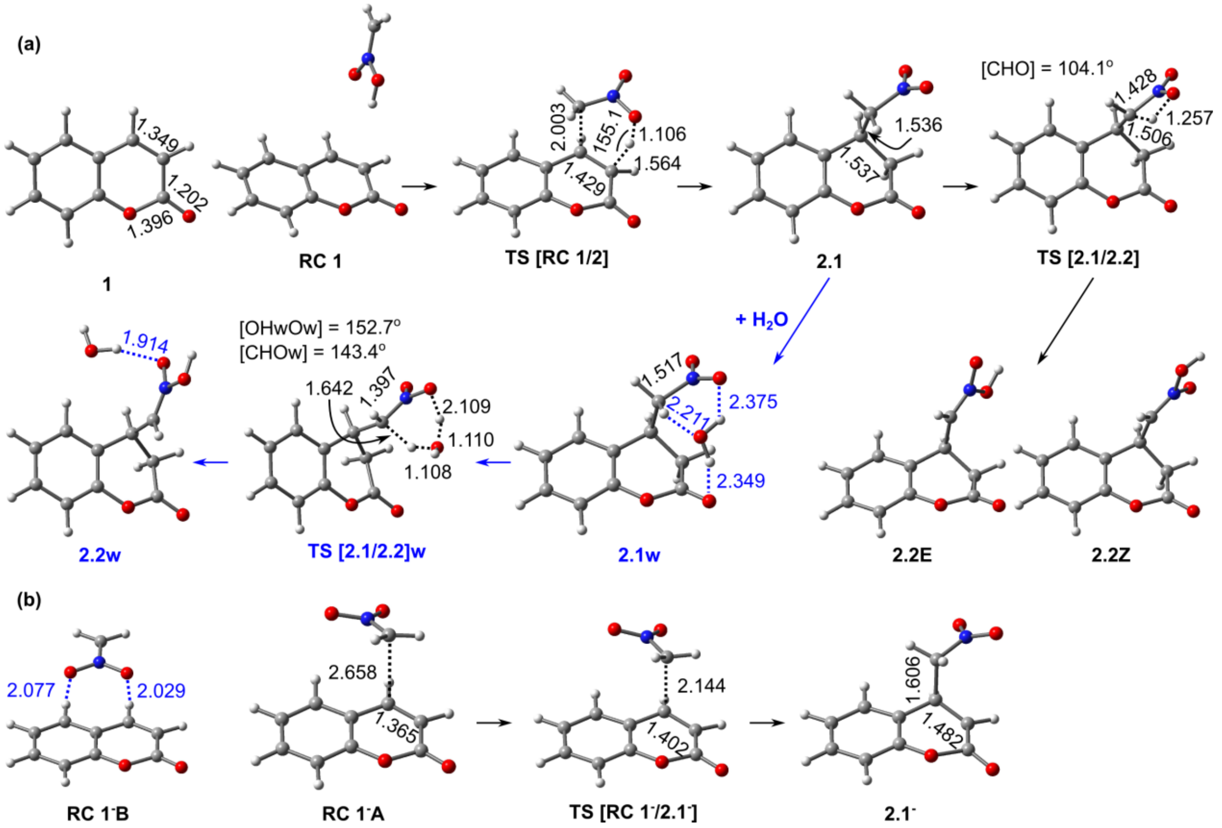

Figure S1. Structures of reaction complexes, transition states and intermediates for Michael addition of nitromethane, CH_3NO_2 (a), and deprotonated nitromethane, $CH_2NO_2^-$ (b), to coumarin **1** (only σ-skeleton is shown; interatomic distances in Å, angles in degrees).

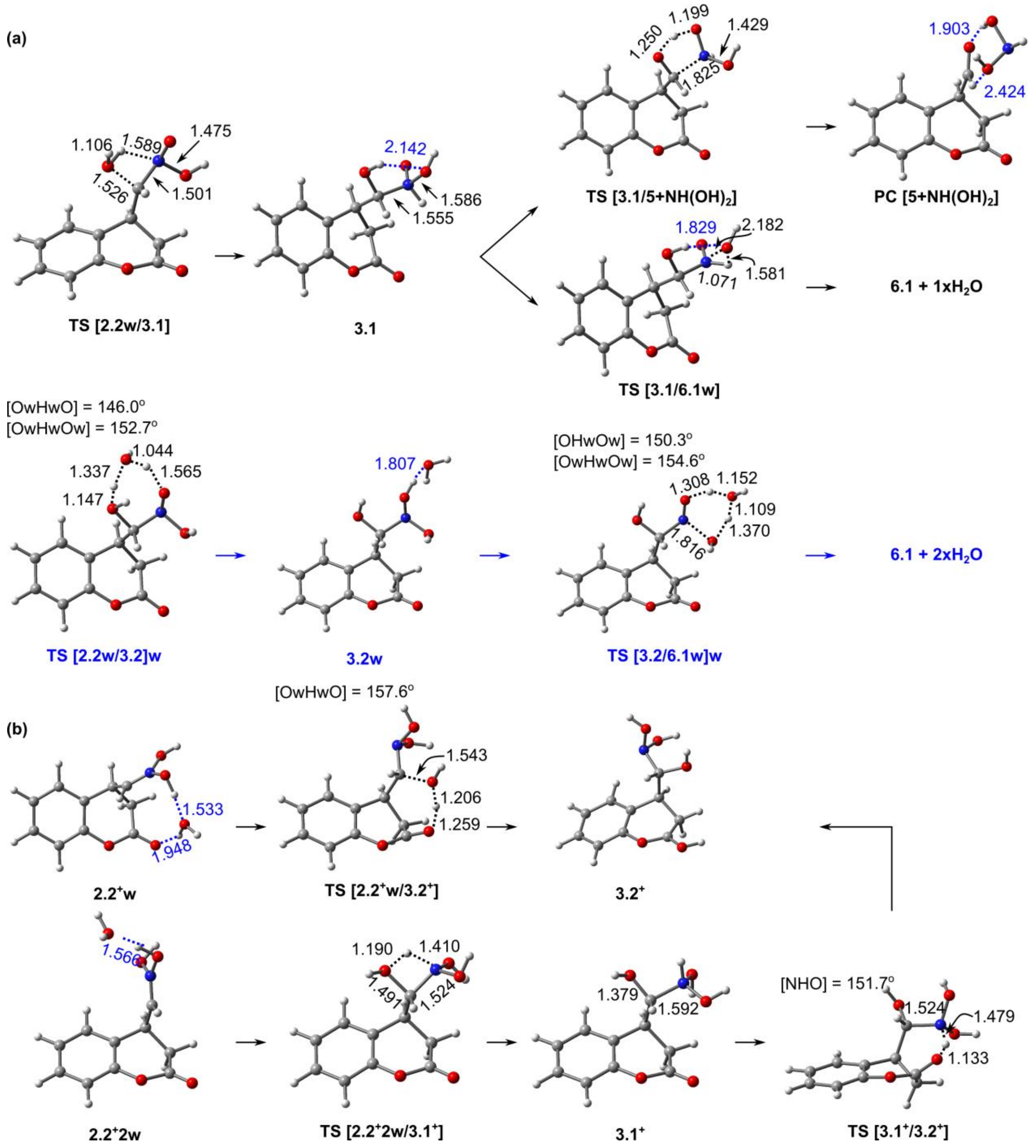

Figure S2. Structures of reaction complexes, transition states and intermediates for O-atom migration assisted by a water molecule (a) and assisted by a water molecule in acidic solution (b). (only σ-skeleton is shown; interatomic distances in Å, angles in degrees).

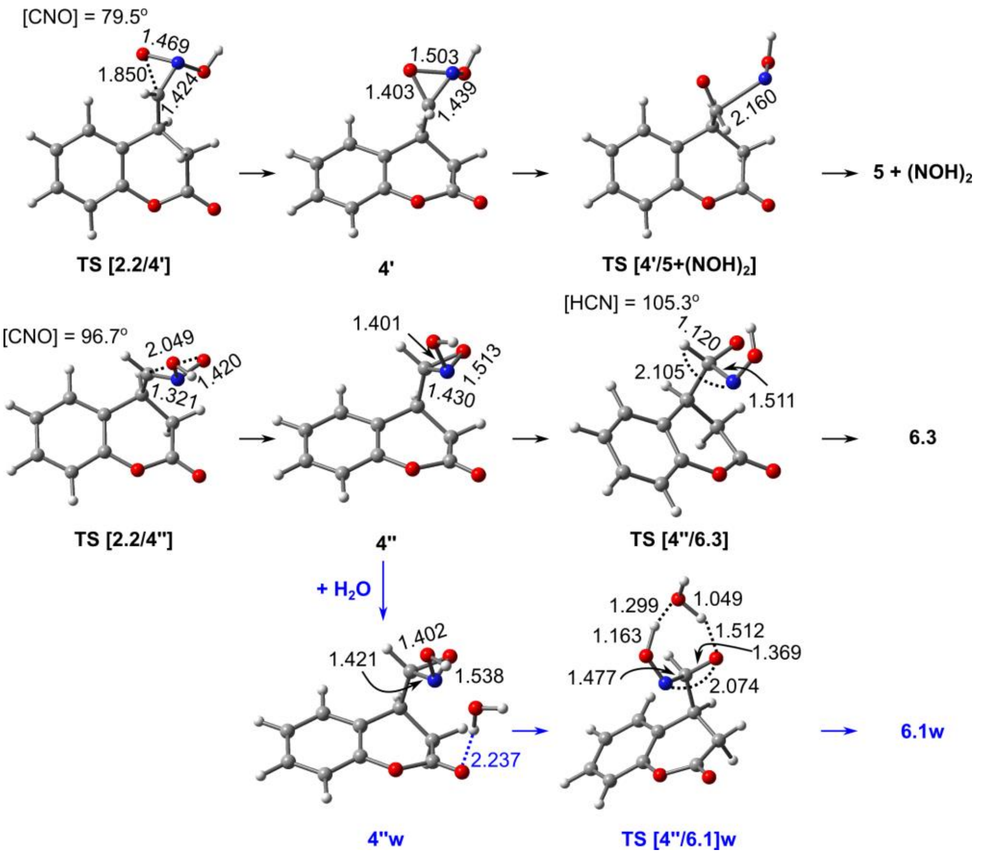

Figure S3. Structures of reaction complexes, transition states and intermediates for O-atom migration via formation of a three-membered oxaziridine cycle. (only σ-skeleton is shown; interatomic distances in Å, angles in degrees).

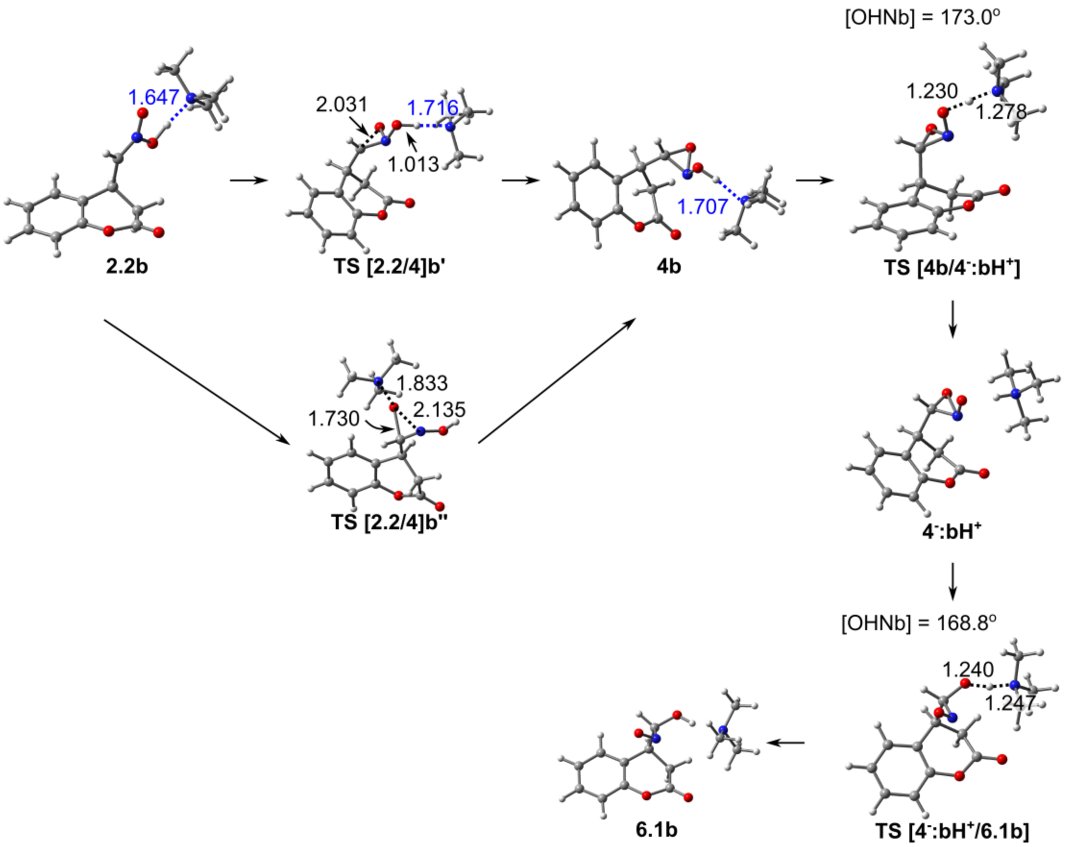

Figure S4. Structures of reaction complexes, transition states and intermediates for O-atom migration via formation of a three-membered oxaziridine cycle, assisted by trimethylamine. (only σ-skeleton is shown; interatomic distances in Å, angles in degrees).

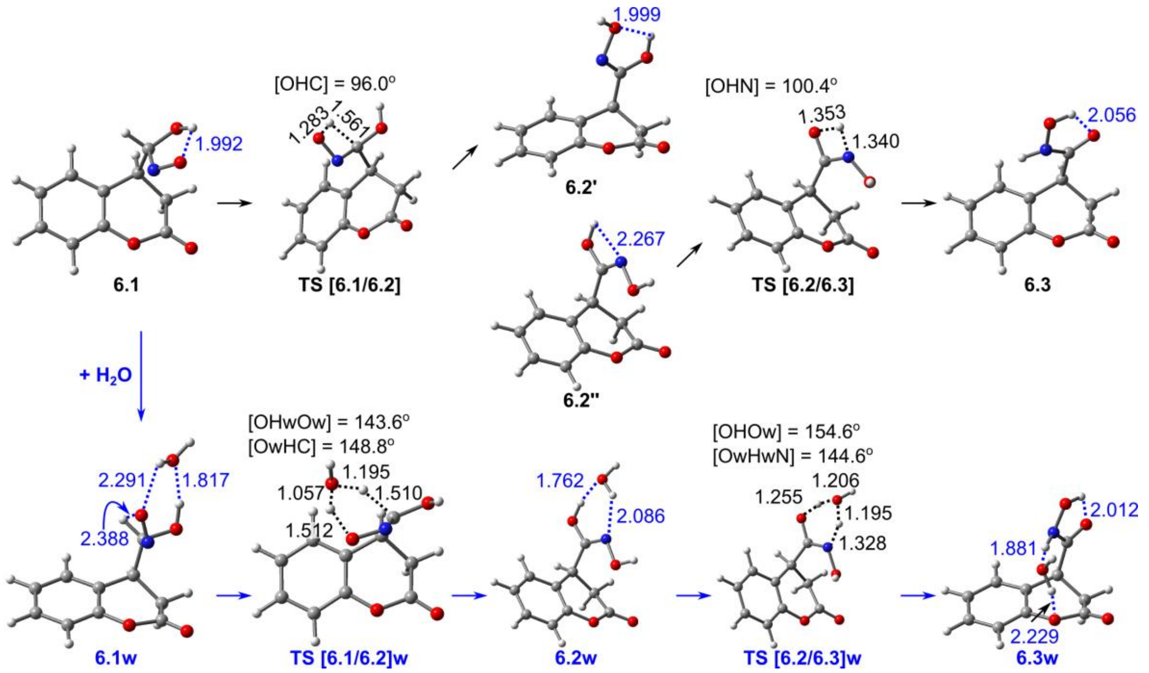

Figure S5. Structures of reaction complexes, transition states and intermediates for the transformation between different tautomeric forms of the nitrosohydroxymethyl group in intermediate **6**. (only σ-skeleton is shown; interatomic distances in Å, angles in degrees).

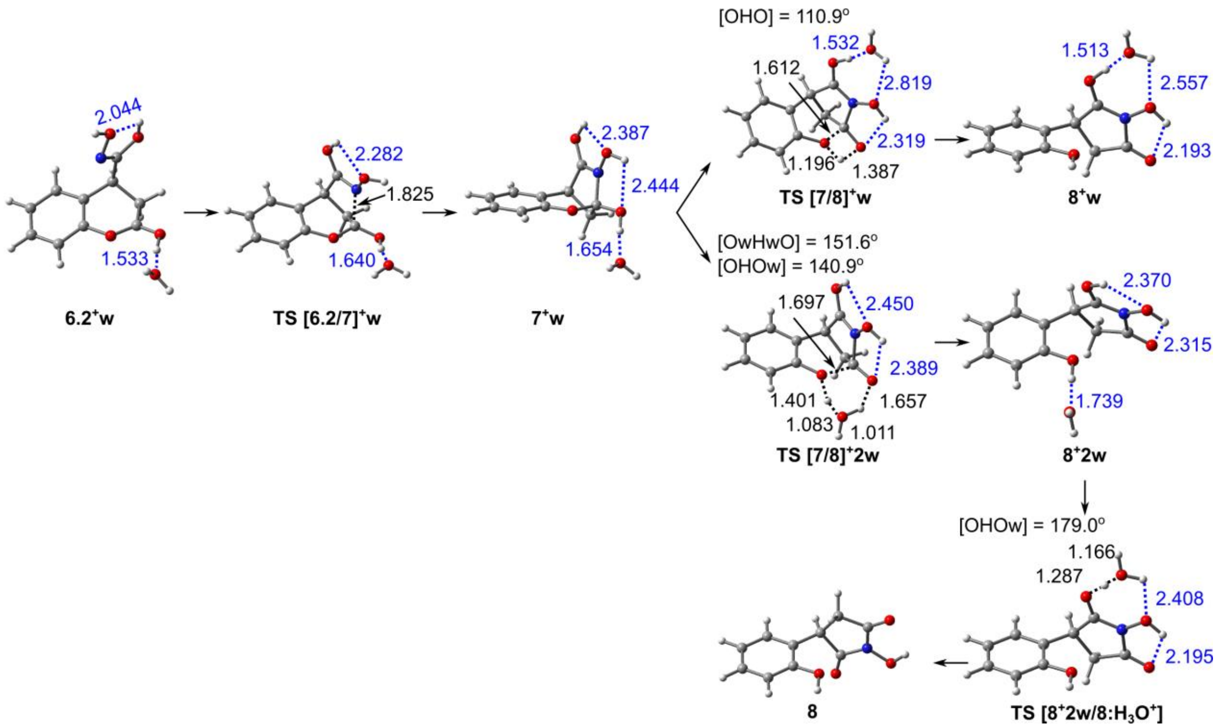

Figure S6. Structures of reaction complexes, transition states and intermediates for formation of pyrrolidine ring from intermediate **6.2**. (only σ-skeleton is shown; interatomic distances in Å, angles in degrees).

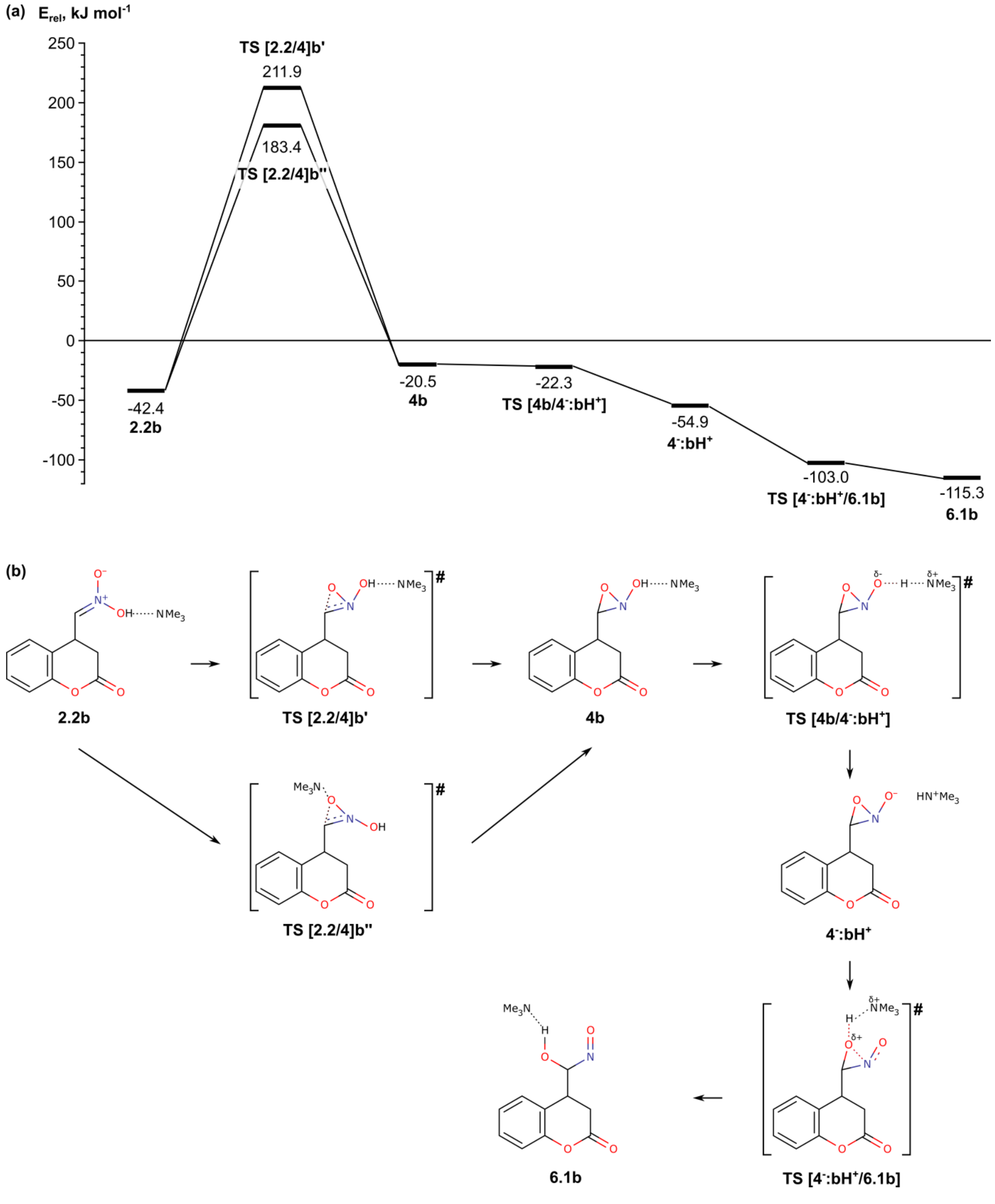

Figure S7. (a) Energy diagram of the mechanism for O-atom migration assisted by trimethylamine. (b) Schematic representation of the reaction paths.

Tables

Table S1. Reaction and activation energies, E_{rea} and E_{act} (in kJ/mol), for various reaction steps calculated as single point energy at MP2/6-311+G* level accounting the solvent effect by PCM. The corrections for the zero-point vibrational energy, ZPE (in kJ/mol), and entropy corrections, S_{tot} and S_{vib}, are obtained from frequency calculations at B3LYP/6-311+G* level. T is equal to 298.15 K.

Structure	E_{rea} or E_{act}	ZPE	T*S_{tot}	T*S_{vib}	ZPE-TΔS	E+ZPE-TΔS
**Michael addition**						
**Neutral system**						
TS [RC 1/2]	69.6	-5.3	-25.5	-24.5	20.1	89.7
2.1	-166.7	13.3	-20.0	-19.1	33.2	-133.4
TS [2.1/2.2]	307.6	-17.1	-3.4	-3.4	-13.7	293.9
2.2E	97.3	-3.7	-0.5	-0.6	-3.3	94.0
2.2Z	90.0	-4.0	-0.5	-0.5	-3.5	86.5
TS [2.1/2.2]w	197.8	-14.6	-13.1	-13.1	-1.5	196.3
2.2w	88.9	-3.5	1.5	1.2	-5.0	83.9
**Negatively charged system**						
TS [RC 1-/2.1-]	21.7	1.8	-14.7	-13.9	16.4	38.2
2.1-	-46.1	6.3	-14.9	-14.1	21.2	-24.9
**Oxygen migration**						
**Neutral system**						
TS [2.2w/3.1]	241.9	-6.9	-14.5	-14.1	7.6	249.5
3.1	16.6	11.1	-13.5	-13.0	24.5	41.1
TS [3.1/6.1w]	73.5	-26.1	-12.7	-12.8	-13.4	60.1
TS [3.1/5+NH(OH)2]	58.7	-12.6	-6.4	-6.5	-6.2	52.5
6.1w	-104.2	-11.6	12.3	12.1	-23.9	-128.2
TS [2.2w/3.2]w	130.8	58.1	-22.3	-22.8	80.5	211.3
3.2w	-36.6	73.9	7.0	6.3	66.8	30.3
TS [3.2/6.1w]w	142.4	-9.0	-30.0	-29.8	21.1	163.5
**Protonated system**						
TS [2.2+2w/3.1+]	149.2	7.1	-10.2	-9.4	17.3	166.6
3.1+	1.3	15.2	-19.0	-18.2	34.2	35.4
TS [3.1+/3.2+]	90.1	-13.7	-5.6	-5.5	-8.2	81.9
TS [2.2+w/3.2+]	101.5	-1.0	-20.6	-20.4	19.6	121.2
3.2+	27.0	9.9	-7.6	-7.5	17.5	44.5
**Nef reaction**						
TS [2.2/4']	164.0	-6.9	-6.0	-6.0	-0.8	163.2
TS [2.2/4"]	261.8	-8.4	-1.3	-1.0	-7.1	254.8
4'	35.5	-0.2	-2.7	-2.5	2.5	38.0
4"	29.0	0.0	-2.7	-2.5	2.7	31.8
TS [4'/5+(NOH)2]	149.5	-8.5	-4.8	-5.0	-3.6	145.8
TS [4"/6.3]	151.8	-8.3	-2.0	-1.7	-6.3	145.5

TS [4''/6.1]w	99.5	-10.6	-3.8	-3.6	-6.8	92.6
6.3	-220.8	2.1	3.0	3.1	-0.9	-221.7
**Base assisted Nef reaction**						
TS [2.2/4]b'	254.3	-6.6	-0.4	0.1	-6.2	248.1
TS [2.2/4]b''	225.8	-2.0	-8.9	-8.3	6.9	232.7
4b	21.8	0.3	-1.0	-0.6	1.3	23.2
TS [4b/4-:bH+]	-1.8	-9.1	-5.9	-5.7	-3.1	-4.9
4-:bH+	-34.4	3.7	-21.9	-21.7	25.7	-8.7
TS [4-:bH+/6.1b]	-48.1	-14.2	19.5	19.8	-33.7	-81.8
TS [4-:bH+/6.2-:bH+]	276.8	-17.2	18.0	17.7	-35.2	241.5
6.1b	-60.4	-5.0	25.5	25.7	-30.5	-90.9
**Tautomerisation of 6**						
TS [6.1/6.2]	335.9	-19.6	-11.2	-11.3	-8.5	327.4
6.2'	-88.4	3.8	0.9	0.8	2.9	-85.5
6.2''	-69.2	4.3	-0.9	-0.8	5.3	-63.9
TS [6.2/6.3]	174.5	-17.3	-6.3	-6.3	-11.0	163.4
6.3	-39.6	-0.8	3.2	3.0	-4.1	-43.6
TS [6.1/6.2]w	178.4	-27.1	-28.2	-27.7	1.1	179.5
6.2w	-79.9	4.8	-5.5	-5.4	10.3	-69.7
TS [6.2/6.3]w	87.4	-31.7	-22.9	-22.6	-8.9	78.6
6.3w	-37.8	-1.4	2.3	2.6	-3.8	-41.6
**Cyclization**						
TS [6.2/7]+w	11.9	-1.5	-16.4	-16.0	15.0	26.9
7+w	-2.1	3.7	-9.1	-8.6	12.8	10.7
TS [7/8]+w	199.4	-15.5	-24.1	-23.8	8.5	208.0
TS [7/8]+2w	84.8	-7.9	-9.8	-9.6	1.9	86.8
8+w	-36.2	-2.0	-8.4	-8.4	6.4	-29.8
8+2w	-18.6	-4.6	10.3	10.0	-14.9	-33.5
TS [8+2w/8:H3O+]	-12.6	-11.4	-5.9	-5.8	-5.5	-18.1
8	-62.9	-96.8	-22.6	-21.3	-74.2	-137.2

[a] Activation energy, E_{act}, and reaction energy, E_{rea}, of the corresponding reaction step.
[b] In entropy S_{tot} all degrees of freedom are included, while in S_{vib} only vibrational degrees of freedom are taken into account.

Table S2. Reaction and activation energies, E_{rea} and E_{act} (in kJ/mol), for various reaction steps in the case of the *ethyl ester of 3-coumarin-carboxylic acid* calculated as single point energy at MP2/6-311+G* level accounting the solvent effect by PCM.

**Structure**	**Erel** [a]	***Erea*** [b] **Eact** [b]
I. Michael addition		
**2.1**	-88.7	-
**TS [2.1/2.2]**	218.0	306.7
**2.2E**	5.8	*94.5*
**2.2Z**	-1.4	*87.3*
**2.1w**	-107.4	
**TS [2.1/2.2]w**	87.4	194.8
**2.2w**	-	-
II. Oxygen migration		
Protonated system		
**2.2^{+}w**	58.5	
**2.2^{+}2w**	50.1	
**TS [2.2^{+}2w/3.1^{+}]**	208.4	158.4
**3.1^{+}**	62.0	*11.9*
**TS [3.1^{+}/3.2^{+}]**	164.1	102.1
**TS [2.2^{+}w/3.2^{+}]**	144.7	86.2
**3.2^{+}**	90.1	*31.6*
Nef reaction		
**TS [2.2/4']**	277.1	271.4
**TS [2.2/4'']**	264.9	266.3
**4'**	43.8	*38.1*
**4''**	31.2	*32.6*
Tautomerisation of 6		
**6.1**	-80.7	
**TS [6.1/6.2]**	252.5	333.2
**6.2'**	-171.1	*-90.3*
**6.2''**	-146.4	*-65.7*
**TS [6.2/6.3]**	24.9	171.3
**6.3**	-190.7	*-44.2*
**6.1w**	-104.2	
**TS [6.1/6.2]w**	69.9	174.1
**6.2w**	-182.5	*-78.2*
**TS [6.2/6.3]w**	-94.9	87.5
**6.3w**	-223.5	*-41.0*
III. Cyclization		
**6.2^{+}w**	-108.0	
**TS [6.2/7]$^{+}$w**	-116.1	-
**7^{+}w**	-116.8	*-8.8*
**TS [7/8]$^{+}$w**	85.1	201.9

**TS [7/8]$^+$2w**	-49.9	67.0
**8$^+$w**	-	-
**8$^+$2w**	-123.9	*-7.1*
**TS [8$^+$2w/8:H_3O^+]**	-134.4	-
**8**	-199.4	*-75.5*

[a] Relative energy of the species, E_{rel}, with respect to the energy of **1** and nitromethane at the corresponding level.

[b] Activation energy, E_{act}, and reaction energy, E_{rea}, of the corresponding reaction step.

Description of the results obtained for the mechanism of the Nef rearrangement assisted by triethylamine

In recent experimental studies, we showed that the final product of the new rearrangement, pyrrolidinedion derivative, could be synthesized also in basic media in presence of triethylamine. By this reason, we modeled O-migration, in which such a base (with trimethymamine as a model) can influence the process of oxygen migration in the three-membered transition state **TS [2.2/4]b** (Fig. S4, S7):

- forming hydrogen bonds between the NOH group and the base in the transition state **TS [2.2/4]b'**;
- coordination of the nitrogen atom from the amine to the migrating oxygen atom from CH_2NO_2H in the transition state **TS [2.2/4]b"**.

The former approach is based on the basic properties of trialkylamine, while the latter one is connected with the possible formation of trialkylamino N-oxide. The formation of hydrogen bond stabilizes the starting compound **2.2b** by 57.1 kJ mol^{-1} with respect to **2.2Z**, while the stabilization of the transition state **TS [2.2/4]b'** is weaker and the energy barrier of the reaction step, 254.3 kJ mol^{-1}, increases compared to the reaction without amine, 167.5 (164.1) kJ mol^{-1}. The transfer of O atom coordinated to N atom from Me_3N through **TS [2.2/4]b"** has a slightly lower energy barrier, 225.8 kJ mol^{-1}, which is still by ~ 60 kJ mol^{-1} higher than the barrier of the reaction without amine.

The reaction step via the presumed transition state **pseudoTS [4b/4$^-$:bH$^+$]** (which has lower energy than the preceding intermediate) instead of the neutral complex **4b** spontaneously leads to formation of the ionic couple **4$^-$:bH$^+$**, in which the NOH group of **4** is deprotonated and the amine is protonated. The formation of the ionic couple leads to the opening of the three-centered ring and complete migration of the oxygen atom to the carbon atom, which was not possible to achieve in absence of the base (see above). The intermediate **6.1** can be easily obtained from **[4]$^-$:bH$^+$** by reverse protonation of **4$^-$** from the protonated base. This process is spontaneous via the structure denoted as **TS [4$^-$:bH$^+$/6.1b]** (Figs. S4, S7).

**Intermediate**	E_{rel} [a]		E_{act} [b] or E_{rea} [b]	
**Transition state**	**L1**	**L2|PCM**	**L1**	**L2|PCM**
Base assisted Nef reaction				
**2.2b**	-9.4	-42.4		
**TS [2.2/4]b'**	234.6	211.9	243.9	254.3
**TS [2.2/4]b''**	232.9	183.4	242.3	225.8
**4b**	36.3	-20.5	45.6	21.8

**pseudoTS [4b/4$^-$:bH$^+$]**	49.2	-22.3	12.9	-1.8
**4$^-$:bH$^+$**	37.5	-54.9	1.2	-34.4
**pseudoTS [4$^-$:bH$^+$/6.1b]**	-24.6	-103.0	-62.1	-48.1
**TS [4$^-$:bH$^+$/6.2$^-$:bH$^+$]**	323.3	221.8	285.8	276.8
**6.1b**	-49.0	-115.3	-86.4	-60.4

[a] Relative energy of the species, E_{rel}, with respect to the energy of **1** and nitromethane at the corresponding level.

[b] Activation energy, E_{act}, and reaction energy, E_{rea}, of the corresponding reaction step.

TS structures (Energies at L1)

**TS [RC 1/2]**			
-742.1602358	ImgFreq/IR Inten: -851.5222/12.3334		
O	-0.91305	1.77920	-0.37935
C	0.29198	2.09008	0.26751
O	0.62625	3.24331	0.28722
C	1.03737	0.96203	0.81543
C	0.49231	-0.35913	0.81706
C	-0.91502	-0.50894	0.41735
C	-1.64923	-1.68757	0.61692
H	-1.17036	-2.53408	1.10083
C	-2.97341	-1.77691	0.21294
H	-3.53217	-2.69128	0.37821
C	-3.58424	-0.67988	-0.40529
H	-4.61850	-0.74483	-0.72586
C	-2.88090	0.50124	-0.60122
H	-3.33971	1.36916	-1.06033
C	-1.55200	0.58382	-0.18457
C	1.38266	-1.44969	-0.60819
H	0.90799	-1.05713	-1.49523
N	2.68510	-1.06404	-0.45595
O	2.90174	0.19732	-0.84144
O	3.52146	-1.65182	0.22209
H	1.76679	1.23321	1.57142
H	0.83484	-1.01504	1.61442
H	1.19675	-2.48151	-0.34092
H	2.17026	0.77539	-0.24679
**TS [2.1/2.2]**			
-742.1354092	ImgFreq/IR inten: -2172.6740/541.3399		
O	-0.93507	1.76505	-0.38671
C	0.26798	2.03500	0.22386
O	0.81958	3.06676	-0.02672
C	0.76377	0.98993	1.20026
H	1.81734	1.19002	1.39487
H	0.22714	1.13886	2.14496
C	0.51107	-0.44375	0.69795
H	0.73051	-1.13868	1.51859
C	-0.95024	-0.56222	0.32723
C	-1.68598	-1.74050	0.47044
H	-1.19747	-2.62198	0.87642
C	-3.02946	-1.79540	0.10852
H	-3.58639	-2.71823	0.22826
C	-3.65518	-0.65702	-0.40127
H	-4.70202	-0.68998	-0.68358
C	-2.94243	0.52875	-0.54609

H	-3.40495	1.42712	-0.93846
C	-1.59919	0.56355	-0.18255
C	1.38161	-0.78999	-0.49738
O	3.38314	-1.17242	0.76573
N	2.77604	-0.96737	-0.24740
O	3.36489	-0.73586	-1.41052
H	1.04620	-1.64942	-1.08034
H	2.20515	-0.32454	-1.66911
**TS [2.1/2.2]w**			
-818.6264588	ImgFreq/IR inten: -1393.6794/102.5452		
O	1.14768	1.75540	0.29575
C	0.03690	2.05236	-0.46764
O	-0.50648	3.10406	-0.29021
C	-0.37106	1.00513	-1.47838
H	-1.38849	1.22155	-1.79920
H	0.27915	1.12046	-2.35426
C	-0.22315	-0.42561	-0.92521
H	-0.37086	-1.12202	-1.75860
C	1.18395	-0.57685	-0.39264
C	1.89989	-1.77497	-0.44065
H	1.43425	-2.64880	-0.88758
C	3.19439	-1.85896	0.06656
H	3.73643	-2.79712	0.01763
C	3.79179	-0.73031	0.62921
H	4.80139	-0.78550	1.02226
C	3.09859	0.47503	0.68333
H	3.54039	1.36748	1.11180
C	1.80437	0.53731	0.17447
C	-1.22917	-0.74303	0.16940
O	-2.90162	-0.90904	-1.40709
N	-2.53355	-1.01985	-0.24705
O	-3.40185	-1.31705	0.68610
H	-0.91988	-1.47335	0.91370
H	-1.72260	0.25790	1.37339
H	-3.10688	-0.41850	1.69101
O	-2.52133	0.41952	2.12461
H	-2.19818	0.23309	3.01520
**TS [RC 1$^-$/2.1$^-$]**			
-741.6739479	ImgFreq/IR inten: -271.0039/625.5705		
O	-2.30114	-0.60008	0.39959
C	-1.86759	-1.89151	-0.03477
O	-2.67164	-2.79038	0.14735
C	-0.58470	-1.94884	-0.62933
C	0.29418	-0.85764	-0.68196
C	-0.28475	0.46315	-0.41935
C	0.38019	1.67012	-0.68385

H	1.38970	1.63524	-1.07700
C	-0.22082	2.89416	-0.41517
H	0.31508	3.81607	-0.61809
C	-1.50695	2.93111	0.13184
H	-1.98143	3.88438	0.34799
C	-2.18730	1.74911	0.40070
H	-3.18895	1.75005	0.81756
C	-1.57981	0.52119	0.12351
C	1.78323	-1.07227	0.84527
H	1.35299	-0.64073	1.73858
N	2.89423	-0.39996	0.36870
O	3.57808	-0.94457	-0.54179
O	3.11813	0.78186	0.74273
H	-0.26915	-2.92858	-0.96867
H	1.11813	-0.89708	-1.38962
H	1.86258	-2.14574	0.76395
**TS [2.2w/3.1]**			
-818.5707078	ImgFreq/IR inten: -811.7078/529.1724		
O	-1.23896	1.76540	-0.60328
C	-0.09207	2.24717	-0.00874
O	0.32308	3.31461	-0.35253
C	0.53024	1.36696	1.05444
H	-0.00419	1.56128	1.99130
H	1.56373	1.67220	1.19691
C	0.41234	-0.13677	0.73799
H	0.71917	-0.70413	1.62138
C	-1.03414	-0.43836	0.40860
C	-1.66596	-1.63913	0.74200
H	-1.09841	-2.41116	1.25259
C	-3.00523	-1.85515	0.43154
H	-3.47863	-2.79358	0.69857
C	-3.73523	-0.85901	-0.21855
H	-4.77985	-1.01902	-0.46299
C	-3.13062	0.34979	-0.54698
H	-3.67624	1.14455	-1.04249
C	-1.79045	0.54762	-0.22802
C	1.36866	-0.50816	-0.38946
N	2.76579	-0.90564	-0.01262
O	3.24154	-0.00877	1.05793
O	3.55772	-0.85650	-1.06593
H	1.35100	0.12339	-1.27696
H	4.06540	0.30857	0.64899
O	1.05941	-1.93234	-0.84229
H	1.36676	-2.01521	-1.76487
H	1.92324	-2.23647	-0.22220

**TS [3.1/6.1]w**			
-818.6414856	ImgFreq/IR inten: -752.8107/81.5313		
O	-1.35687	1.72360	-0.62448
C	-0.24951	2.26070	-0.02374
O	0.16926	3.31463	-0.40952
C	0.33810	1.45447	1.11586
H	-0.28782	1.63583	1.99693
H	1.32248	1.86363	1.34019
C	0.34118	-0.05841	0.83804
H	0.62200	-0.59102	1.74969
C	-1.05746	-0.45917	0.42311
C	-1.61125	-1.71043	0.70427
H	-1.00502	-2.44724	1.21961
C	-2.91163	-2.01893	0.31754
H	-3.32491	-2.99607	0.54203
C	-3.67881	-1.07066	-0.36074
H	-4.69340	-1.30475	-0.66483
C	-3.14701	0.18102	-0.65205
H	-3.71824	0.93520	-1.18094
C	-1.84432	0.47039	-0.25951
C	1.35147	-0.47925	-0.23870
N	2.77682	-0.03210	0.18164
O	3.57954	-1.10807	-1.53866
O	3.17865	-0.20706	1.30294
H	1.21402	0.05631	-1.18605
H	4.41599	-1.53966	-1.31310
O	1.34422	-1.84391	-0.39229
H	2.08115	-2.02235	-1.02591
H	3.43293	0.18294	-0.63743

**TS [3.1/5+NH(OH)$_2$]**			
-818.6452361	ImgFreq/IR inten: -1142.8770/826.7598		
O	-1.39874	1.79977	-0.42400
C	-0.23620	2.23703	0.14844
O	0.17411	3.32826	-0.12640
C	0.42079	1.29042	1.13443
H	-0.09382	1.42689	2.09273
H	1.44305	1.64600	1.27278
C	0.32466	-0.18992	0.72980
H	0.60685	-0.82219	1.57865
C	-1.11253	-0.49242	0.36328
C	-1.69101	-1.75274	0.53364
H	-1.08263	-2.55805	0.93057
C	-3.01805	-1.98365	0.18555
H	-3.44934	-2.96918	0.32305
C	-3.78859	-0.94677	-0.34256
H	-4.82423	-1.11929	-0.61543

C	-3.23210	0.31488	-0.52319
H	-3.80448	1.13784	-0.93546
C	-1.90244	0.52534	-0.17245
C	1.25197	-0.57052	-0.43784
N	2.94728	-0.38843	0.21179
O	3.66053	0.48563	-0.66475
O	3.40616	-1.63819	0.18479
H	1.31960	0.18398	-1.23338
H	4.49569	0.01432	-0.82750
O	1.30268	-1.81604	-0.78827
H	2.45192	-2.09338	-0.38127
H	3.04947	0.00680	1.14707
**TS [2.2w/3.2]w**			
-895.0662618	ImgFreq/IR inten: -784.3438/640.9200		
O	-1.83800	1.63691	-0.45776
C	-0.84715	2.30122	0.23886
O	-0.70208	3.47054	0.04190
C	-0.05150	1.47029	1.22423
H	-0.62514	1.45038	2.15826
H	0.88241	1.99263	1.41599
C	0.15977	0.01549	0.76338
H	0.56903	-0.55063	1.60653
C	-1.18226	-0.55699	0.36283
C	-1.54612	-1.89059	0.56307
H	-0.83675	-2.57003	1.02598
C	-2.80007	-2.35815	0.18114
H	-3.06404	-3.39730	0.34427
C	-3.71400	-1.48448	-0.40928
H	-4.69348	-1.84065	-0.71003
C	-3.37825	-0.14924	-0.60677
H	-4.07190	0.55314	-1.05439
C	-2.11983	0.29989	-0.21650
C	1.18725	-0.07822	-0.36449
N	2.56259	0.32998	0.05459
O	2.62278	1.74601	-0.17236
O	3.44785	-0.28516	-0.83293
H	0.88094	0.44140	-1.27924
H	3.02925	1.81289	-1.05662
O	1.35272	-1.48937	-0.76321
H	2.08864	-1.35379	-1.43341
H	2.01352	-2.02178	0.00889
H	3.65624	-1.55583	0.05575
O	3.19966	-2.37470	0.51533
H	3.32267	-2.31942	1.46920

**TS [3.2/6.1w]w**				
-895.0882589	ImgFreq/IR inten: -1326.5756/194.2267			
O		-1.48825	1.78215	-0.57039
C		-0.31750	2.11766	0.06492
O		0.28456	3.08227	-0.31896
C		0.09435	1.20919	1.20043
H		-0.56392	1.42516	2.05020
H		1.11430	1.45374	1.47898
C		-0.08867	-0.27162	0.82358
H		0.11606	-0.89007	1.70152
C		-1.53610	-0.44677	0.40838
C		-2.28467	-1.59609	0.66944
H		-1.80269	-2.43350	1.16200
C		-3.62265	-1.68059	0.29480
H		-4.18707	-2.58274	0.50497
C		-4.23391	-0.60488	-0.35052
H		-5.27672	-0.66395	-0.64391
C		-3.50904	0.55193	-0.61821
H		-3.95660	1.40341	-1.11802
C		-2.17304	0.61561	-0.23583
C		0.86782	-0.77131	-0.27462
N		2.29988	-0.85002	0.22790
O		2.84120	0.88298	0.21210
O		3.00879	-1.42818	-0.69996
H		0.85589	-0.14045	-1.17620
H		2.57601	1.28489	-0.63179
O		0.49683	-2.08587	-0.58100
H		1.14618	-2.43834	-1.20610
H		4.21485	-1.08506	-0.32812
O		4.94743	-0.30107	0.09120
H		4.14204	0.45416	0.19975
H		5.61862	-0.03409	-0.54661

**TS [2.2$^+$2w/3.1$^+$]**				
-818.9474032	ImgFreq/IR inten: -1487.8898/1295.6481			
O		-1.11566	1.83089	-0.47678
C		0.04886	2.18125	0.15878
O		0.59509	3.20183	-0.12798
C		0.56187	1.19052	1.19283
H		0.00035	1.34044	2.12032
H		1.59752	1.46111	1.40391
C		0.35415	-0.27268	0.75347
H		0.62309	-0.95370	1.56382
C		-1.10603	-0.44790	0.38187
C		-1.82230	-1.62554	0.62004
H		-1.32576	-2.46890	1.08998

C	-3.16875	-1.71265	0.27948
H	-3.71814	-2.62599	0.47591
C	-3.81183	-0.61630	-0.29855
H	-4.86240	-0.67848	-0.55874
C	-3.11841	0.56721	-0.53262
H	-3.60015	1.43378	-0.96996
C	-1.77326	0.63938	-0.18946
C	1.21741	-0.60487	-0.45022
N	2.70824	-0.85723	-0.26222
O	3.14975	-0.99463	1.04385
O	3.42057	0.12860	-0.94396
H	1.10531	0.06219	-1.30313
H	3.26225	-0.09887	1.41615
O	1.03074	-2.01634	-0.89329
H	0.52177	-2.15688	-1.70936
H	2.21889	-2.04646	-0.83979
H	4.28554	-0.26088	-1.16196
**TS [3.1$^+$/3.2$^+$]**			
-818.9847008	ImgFreq/IR inten: -790.2056/1952.7991		
O	-0.88616	1.83313	-0.36178
C	0.27450	1.75721	0.21731
O	1.28439	2.21150	-0.38698
C	0.41588	0.88812	1.41642
H	-0.37291	1.09468	2.14204
H	1.38492	1.00944	1.88939
C	0.18713	-0.51735	0.80402
H	0.33997	-1.27948	1.56857
C	-1.25950	-0.48516	0.32770
C	-2.13958	-1.56318	0.40072
H	-1.78042	-2.51918	0.76726
C	-3.47772	-1.41404	0.02973
H	-4.14893	-2.26226	0.09742
C	-3.95677	-0.18233	-0.41011
H	-4.99760	-0.06908	-0.68964
C	-3.10033	0.91646	-0.49809
H	-3.44091	1.88186	-0.85311
C	-1.78322	0.73458	-0.12876
C	1.18455	-0.81044	-0.35991
N	2.47555	-0.01764	-0.19098
O	3.33413	-0.29883	-1.24724
O	3.06353	-0.45020	1.02359
H	0.80166	-0.41951	-1.30653
H	3.49187	-1.26396	-1.21847
O	1.47596	-2.16603	-0.45459
H	1.21921	-2.52498	-1.31264
H	2.06745	1.40283	-0.25442

H	3.86240	0.09337	1.12889
**TS [2.2⁺w/3.2⁺]**			
-818.9805439	ImgFreq/IR inten: -1126.4281/2592.9207		
O	-1.40383	1.81772	-0.30938
C	-0.22882	2.05621	0.21438
O	0.58681	2.76695	-0.40433
C	0.19405	1.23805	1.39307
H	-0.58407	1.21746	2.15753
H	1.11336	1.62393	1.82670
C	0.32699	-0.17677	0.78394
H	0.64066	-0.86778	1.56517
C	-1.07151	-0.53019	0.29225
C	-1.59882	-1.82074	0.32963
H	-0.97016	-2.64309	0.65525
C	-2.92665	-2.05561	-0.02792
H	-3.32096	-3.06438	0.00893
C	-3.74906	-0.99981	-0.41574
H	-4.78288	-1.18336	-0.68408
C	-3.24973	0.30184	-0.46528
H	-3.86218	1.13851	-0.77981
C	-1.92895	0.50270	-0.11402
C	1.40460	-0.21815	-0.33980
N	2.24356	-1.36803	-0.24042
O	2.87272	-1.47424	1.00485
O	3.22626	-1.21078	-1.24854
H	0.97946	-0.21240	-1.34147
H	3.52669	-0.75330	1.08057
H	3.44291	-2.10641	-1.54853
O	2.28222	1.04891	-0.27308
H	2.94264	0.99603	-0.98604
H	1.62353	2.05728	-0.33038
**TS [2.2/4']**			
-742.1531741	ImgFreq/IR inten: -106.9246/73.8740		
O	1.63521	1.67808	-0.23923
C	0.48239	2.35135	0.05631
O	0.43611	3.53451	-0.11618
C	-0.65024	1.52364	0.62899
H	-1.57025	2.09403	0.51745
H	-0.45003	1.40975	1.70220
C	-0.75716	0.12878	-0.04307
H	-1.11317	0.25933	-1.06771
C	0.60883	-0.52709	-0.03366
C	0.80697	-1.90988	0.01753
H	-0.05697	-2.56481	0.04224
C	2.09190	-2.44706	0.01218

H	2.22573	-3.52256	0.04958
C	3.19987	-1.60257	-0.04818
H	4.20306	-2.01508	-0.05300
C	3.02388	-0.22403	-0.11178
H	3.86513	0.45665	-0.17357
C	1.73459	0.29653	-0.10691
C	-1.78458	-0.63916	0.70611
N	-3.15396	-0.72851	0.32624
O	-3.40095	0.25041	-0.68479
O	-2.62327	-1.94979	-0.29455
H	-4.36318	0.23477	-0.77085
H	-1.57817	-1.06110	1.68295
**TS [2.2/4'']**			
-742.1249367	ImgFreq/IR inten: -914.2135/43.8485		
O	0.49836	1.65963	0.58338
C	-0.63987	1.89507	-0.17301
O	-1.31415	2.83917	0.10627
C	-0.88889	0.94736	-1.32687
H	-0.33674	1.34084	-2.18776
H	-1.95145	0.97078	-1.55856
C	-0.41206	-0.48333	-1.05771
H	-0.42184	-1.05413	-1.99385
C	1.00522	-0.45683	-0.50415
C	1.94641	-1.46274	-0.74056
H	1.67345	-2.31012	-1.36325
C	3.22443	-1.38371	-0.19750
H	3.94666	-2.16853	-0.39269
C	3.57244	-0.28610	0.59289
H	4.56745	-0.21657	1.01912
C	2.65223	0.72771	0.83354
H	2.90141	1.59199	1.43822
C	1.37379	0.63387	0.28742
C	-1.23512	-1.25756	-0.07698
N	-2.30925	-0.76228	0.51158
O	-2.73606	-1.55987	1.59977
O	-3.15332	-0.79610	-0.63028
H	-3.28990	-0.96359	2.12412
H	-0.87442	-2.23961	0.23810
**TS [4'/5+(NOH)$_2$]**			
-742.1536390	ImgFreq/IR inten: -170.4202/2.0477		
O	1.08385	1.81242	0.34357
C	-0.17588	2.15954	-0.08158
O	-0.59639	3.24504	0.19834
C	-0.90942	1.12950	-0.91333
H	-0.55001	1.24445	-1.94294
H	-1.96677	1.38901	-0.90416

C	-0.66429	-0.31765	-0.46616
H	-1.04241	-1.01040	-1.22145
C	0.82533	-0.53069	-0.28391
C	1.44458	-1.77027	-0.46670
H	0.83615	-2.62441	-0.74655
C	2.81670	-1.91752	-0.28867
H	3.28078	-2.88659	-0.43603
C	3.59062	-0.81571	0.07911
H	4.66115	-0.92224	0.21827
C	2.99401	0.42603	0.27017
H	3.57031	1.29720	0.55978
C	1.61966	0.55426	0.09234
C	-1.36548	-0.66617	0.84794
N	-3.48198	-0.48570	0.45796
O	-3.73104	-0.88929	-0.71862
O	-1.71667	-1.81003	1.13080
H	-4.43835	-1.58056	-0.72399
H	-1.34998	0.12041	1.62329
**TS [4''/6.3]**			
-742.1545510	ImgFreq/IR inten: -593.5718/183.6230		
O	0.30364	1.75450	0.50909
C	-0.89761	1.91598	-0.15770
O	-1.61976	2.80030	0.19533
C	-1.15424	0.99566	-1.33055
H	-0.74045	1.50099	-2.21137
H	-2.23288	0.92727	-1.47034
C	-0.53632	-0.39966	-1.20136
H	-0.51161	-0.86480	-2.19020
C	0.84523	-0.33445	-0.62288
C	1.81640	-1.31855	-0.85527
H	1.57349	-2.15160	-1.50872
C	3.07812	-1.23541	-0.28053
H	3.81709	-2.00447	-0.47702
C	3.39218	-0.15146	0.54576
H	4.37596	-0.07809	0.99697
C	2.45069	0.83963	0.78950
H	2.67024	1.69142	1.42272
C	1.18657	0.74147	0.20868
C	-1.43227	-1.36793	-0.34640
N	-1.31381	-0.87066	1.07574
O	-2.05089	-1.51019	1.89173
O	-2.72942	-1.35119	-0.55024
H	-2.55297	-2.21966	1.39935
H	-0.92324	-2.36543	-0.35427

TS [4''/6.1]w			
-818.6381640	ImgFreq/IR inten: -1006.4768/581.8438		
O	-1.35245	1.57632	-0.65636
C	-0.37710	2.26192	0.03711
O	-0.06970	3.35149	-0.34798
C	0.16608	1.59510	1.28193
H	-0.49665	1.89725	2.10175
H	1.14882	2.01739	1.48599
C	0.22069	0.06232	1.22472
H	0.32745	-0.31266	2.24833
C	-1.04999	-0.48236	0.61801
C	-1.54687	-1.75654	0.91249
H	-1.01235	-2.37765	1.62626
C	-2.71235	-2.23230	0.31977
H	-3.08141	-3.22245	0.56412
C	-3.40602	-1.42419	-0.58271
H	-4.31757	-1.78331	-1.04858
C	-2.93674	-0.15101	-0.88460
H	-3.45848	0.49851	-1.57807
C	-1.76486	0.30796	-0.28601
C	1.46845	-0.46756	0.49673
N	1.53765	0.05668	-0.88240
O	2.22571	-0.62631	-1.68639
O	2.65138	0.11441	0.86665
H	2.97476	-1.34641	-1.16342
H	1.46552	-1.57184	0.50266
O	3.90433	-1.66855	-0.31508
H	4.80486	-1.54244	-0.63560
H	3.63958	-0.91207	0.36201
**TS [2.2/4]b'**			
-916.6616805	ImgFreq/IR inten: -894.6979/67.2922		
O	-1.39132	1.65929	0.22523
C	-0.58332	1.33600	1.30198
O	0.26157	2.11850	1.62393
C	-0.89149	0.03203	2.00234
H	-1.70060	0.23197	2.71431
H	-0.00539	-0.27282	2.55367
C	-1.34823	-1.06949	1.04066
H	-1.73051	-1.92162	1.61397
C	-2.45500	-0.52816	0.14841
C	-3.50394	-1.30897	-0.34383
H	-3.55482	-2.35974	-0.07277
C	-4.48104	-0.75506	-1.16500
H	-5.29134	-1.37221	-1.53704
C	-4.41485	0.59842	-1.50126
H	-5.17341	1.03823	-2.13989

C	-3.38209	1.39371	-1.01675
H	-3.31220	2.44748	-1.26071
C	-2.40823	0.82430	-0.19976
C	-0.27782	-1.59275	0.12976
N	0.95670	-1.12822	0.13236
O	1.70625	-1.48920	-0.97925
O	1.31590	-1.75978	1.37704
H	2.49672	-0.85581	-0.95518
H	-0.56711	-2.29749	-0.65443
N	3.81118	0.22047	-0.71349
C	4.37740	-0.19610	0.57612
C	4.79005	0.08750	-1.79402
C	3.28229	1.58784	-0.64123
H	2.81066	1.85052	-1.59063
H	4.07609	2.32237	-0.43250
H	2.53089	1.65759	0.14590
H	4.32992	0.35948	-2.74649
H	5.12983	-0.94809	-1.86080
H	5.66967	0.73224	-1.63792
H	3.59887	-0.18525	1.34005
H	5.20434	0.45962	0.89061
H	4.75170	-1.21841	0.49771
**TS [2.2/4]b''**			
-916.6623065	ImgFreq/IR inten: -623.7936/365.4912		
O	3.04867	-0.12165	0.84069
C	2.85927	-1.44705	0.50974
O	3.57226	-2.26553	1.01503
C	1.74981	-1.73007	-0.47627
H	1.49152	-2.78241	-0.39193
H	2.14337	-1.55795	-1.48607
C	0.52870	-0.81799	-0.22394
H	0.15738	-1.02962	0.78474
C	1.02147	0.61393	-0.28419
C	0.32992	1.68271	-0.85998
H	-0.64539	1.50020	-1.29446
C	0.87070	2.96793	-0.88056
H	0.31562	3.78085	-1.33728
C	2.12515	3.20437	-0.32071
H	2.55234	4.20157	-0.33171
C	2.84135	2.15326	0.24421
H	3.82476	2.30058	0.67601
C	2.28776	0.87731	0.24854
C	-0.61126	-1.11825	-1.17882
N	-1.16692	-2.31671	-1.32756
O	-0.90302	-3.12603	-0.21193
O	-2.09936	-0.60157	-0.46276

H	-1.46457	-3.89596	-0.35935
H	-0.60052	-0.58036	-2.12286
N	-3.10331	0.55232	0.54768
C	-3.53325	1.65652	-0.30560
C	-4.19966	-0.34201	0.91284
C	-2.30456	0.97107	1.69661
H	-3.79019	-1.20840	1.42905
H	-4.69667	-0.68256	0.00649
H	-4.92422	0.16902	1.56295
H	-2.67232	2.26555	-0.57624
H	-4.26740	2.29259	0.20937
H	-3.97868	1.24970	-1.21215
H	-1.94442	0.08456	2.21703
H	-2.90052	1.57949	2.39126
H	-1.44933	1.55180	1.35488
**TS [4b/4$^-$:bH$^+$]**			
-916.7322648	ImgFreq/IR inten: -860.8006/6016.3728		
O	1.18397	-1.31140	1.06682
C	0.48519	-0.46830	1.90035
O	-0.38370	-0.94334	2.57896
C	0.92689	0.97629	1.91939
H	1.76840	1.04148	2.61989
H	0.10841	1.57028	2.32217
C	1.36394	1.49871	0.54272
H	1.86747	2.46192	0.68166
C	2.31601	0.51387	-0.08774
C	3.32760	0.88299	-0.97867
H	3.45423	1.93407	-1.22262
C	4.17105	-0.06726	-1.54867
H	4.94994	0.24188	-2.23750
C	4.01350	-1.41482	-1.22394
H	4.66856	-2.16256	-1.65861
C	3.01691	-1.80651	-0.33541
H	2.87400	-2.84625	-0.06343
C	2.17926	-0.84229	0.21775
C	0.15395	1.79229	-0.36791
N	-0.86590	0.77344	-0.33508
O	-1.48392	0.61103	-1.46171
O	-0.87687	2.49441	0.19068
H	-2.50880	0.03355	-1.10169
H	0.45349	2.09137	-1.38114
N	-3.54199	-0.51826	-0.59118
C	-3.09648	-1.72614	0.14128
C	-4.14013	0.47872	0.32619
C	-4.45959	-0.85845	-1.69557
H	-4.71686	0.04746	-2.24546

H	-5.37709	-1.32072	-1.31610
H	-3.96593	-1.55284	-2.37636
H	-4.38601	1.38013	-0.23529
H	-3.41229	0.73873	1.09224
H	-5.04718	0.07921	0.79227
H	-2.59300	-2.40031	-0.55248
H	-3.95287	-2.24348	0.58735
H	-2.39374	-1.44051	0.92242
**TS [4$^-$:bH$^+$/6.1]b**			
-916.7603690	ImgFreq/IR inten: -820.5050/5432.8938		
O	1.18397	-1.31140	1.06682
C	0.48519	-0.46830	1.90035
O	-0.38370	-0.94334	2.57896
C	0.92689	0.97629	1.91939
H	1.76840	1.04148	2.61989
H	0.10841	1.57028	2.32217
C	1.36394	1.49871	0.54272
H	1.86747	2.46192	0.68166
C	2.31601	0.51387	-0.08774
C	3.32760	0.88299	-0.97867
H	3.45423	1.93407	-1.22262
C	4.17105	-0.06726	-1.54867
H	4.94994	0.24188	-2.23750
C	4.01350	-1.41482	-1.22394
H	4.66856	-2.16256	-1.65861
C	3.01691	-1.80651	-0.33541
H	2.87400	-2.84625	-0.06343
C	2.17926	-0.84229	0.21775
C	0.15395	1.79229	-0.36791
N	-0.86590	0.77344	-0.33508
O	-1.48392	0.61103	-1.46171
O	-0.87687	2.49441	0.19068
H	-2.50880	0.03355	-1.10169
H	0.45349	2.09137	-1.38114
N	-3.54199	-0.51826	-0.59118
C	-3.09648	-1.72614	0.14128
C	-4.14013	0.47872	0.32619
C	-4.45959	-0.85845	-1.69557
H	-4.71686	0.04746	-2.24546
H	-5.37709	-1.32072	-1.31610
H	-3.96593	-1.55284	-2.37636
H	-4.38601	1.38013	-0.23529
H	-3.41229	0.73873	1.09224
H	-5.04718	0.07921	0.79227
H	-2.59300	-2.40031	-0.55248
H	-3.95287	-2.24348	0.58735

H		-2.39374	-1.44051	0.92242
**TS [4$^-$:bH$^+$/6.2]$^-$:bH$^+$**				
-916.6279092	ImgFreq/IR inten: -1485.9711/2006.5186			
O		-2.06978	1.48418	-0.89267
C		-1.23231	2.16121	-0.03979
O		-0.70629	3.16397	-0.43594
C		-1.11213	1.60333	1.35923
H		-1.97168	1.99022	1.92203
H		-0.21063	2.02012	1.80398
C		-1.11754	0.06377	1.42054
H		-1.28352	-0.23042	2.46290
C		-2.26313	-0.45205	0.58093
C		-2.91958	-1.65727	0.84237
H		-2.59673	-2.25157	1.69187
C		-3.96236	-2.10272	0.03465
H		-4.45815	-3.04199	0.25659
C		-4.36593	-1.33545	-1.05885
H		-5.17916	-1.67198	-1.69370
C		-3.72652	-0.13256	-1.33981
H		-4.01647	0.48262	-2.18420
C		-2.68204	0.29329	-0.52274
C		0.18024	-0.62093	0.99140
N		0.88129	-0.06545	-0.14484
O		1.54764	-1.27662	-0.56881
O		1.38821	0.17782	1.25110
H		3.00039	-0.84830	-0.45486
H		0.93403	-1.77729	0.29112
N		4.00473	-0.42730	-0.36907
C		3.87091	1.04087	-0.59720
C		4.48007	-0.72618	1.00928
C		4.84054	-1.07735	-1.40995
H		4.85349	-2.15279	-1.23694
H		5.85839	-0.68554	-1.37204
H		4.40447	-0.87914	-2.38789
H		4.53574	-1.80675	1.13692
H		3.75274	-0.32050	1.70897
H		5.46553	-0.28348	1.16547
H		3.44333	1.20591	-1.58449
H		4.85345	1.51157	-0.52834
H		3.19319	1.44592	0.15012
**TS [6.1/6.2]**				
-742.1181135	ImgFreq/IR inten: -2359.5993/442.3123			
O		0.42755	1.76403	0.60656
C		-0.77569	2.01984	-0.00956
O		-1.45177	2.91531	0.40569

C	-1.10618	1.16702	-1.21536
H	-0.60007	1.62863	-2.07209
H	-2.17804	1.23782	-1.39116
C	-0.64165	-0.29912	-1.09019
H	-0.68648	-0.74526	-2.08917
C	0.78676	-0.32640	-0.59663
C	1.68196	-1.35222	-0.91545
H	1.34213	-2.16759	-1.54767
C	2.98797	-1.34213	-0.43818
H	3.66759	-2.14678	-0.69691
C	3.41908	-0.28950	0.37254
H	4.43608	-0.27239	0.74970
C	2.54907	0.74359	0.69840
H	2.85746	1.57206	1.32557
C	1.24276	0.71587	0.21341
C	-1.55008	-1.15656	-0.21619
O	-2.92581	-0.97337	-0.54957
N	-1.38468	-0.99295	1.18149
O	-1.75613	-2.09627	1.67493
H	-3.28654	-1.77623	-0.93747
H	-1.77404	-2.53961	0.47121
**TS [6.2/6.3]**			
-742.2090475	ImgFreq/IR inten: -1884.4039/398.1168		
O	-0.12819	-1.83583	0.25735
C	1.05676	-1.79696	-0.43943
O	1.81981	-2.70914	-0.32106
C	1.25744	-0.63030	-1.38393
H	0.86249	-0.95891	-2.35248
H	2.33145	-0.49941	-1.51264
C	0.54203	0.68189	-1.00741
H	0.44453	1.28860	-1.91017
C	-0.84493	0.38349	-0.46810
C	-1.88549	1.31441	-0.53609
H	-1.69463	2.28342	-0.98678
C	-3.14571	1.01614	-0.02870
H	-3.94169	1.75011	-0.08861
C	-3.37914	-0.23100	0.55333
H	-4.35974	-0.47426	0.94830
C	-2.35802	-1.17145	0.62617
H	-2.51389	-2.14879	1.06833
C	-1.09904	-0.85574	0.11969
C	1.29765	1.55358	-0.03733
O	1.40832	2.83195	-0.17108
N	1.92257	1.26709	1.07929
O	2.24011	-0.05191	1.43425
H	2.12097	2.58602	0.95222

H	1.92282	-0.16138	2.34187
**TS [6.1/6.2]w**			
-818.6277658	ImgFreq/IR inten: -1607.8191/738.1699		
O	-0.17672	-1.92095	0.65956
C	1.00489	-2.10932	-0.01706
O	1.77664	-2.93073	0.38556
C	1.19975	-1.25564	-1.25135
H	0.63631	-1.72099	-2.06874
H	2.25402	-1.27360	-1.51368
C	0.67100	0.18514	-1.05687
H	0.70638	0.68574	-2.02832
C	-0.76489	0.07350	-0.60191
C	-1.78192	0.92900	-1.03225
H	-1.53290	1.75804	-1.68687
C	-3.09855	0.74325	-0.62041
H	-3.87316	1.42251	-0.95972
C	-3.41726	-0.32270	0.22193
H	-4.44058	-0.47390	0.54874
C	-2.42632	-1.20719	0.63429
H	-2.64703	-2.05172	1.27676
C	-1.11675	-1.00596	0.21205
C	1.65049	0.95176	-0.12461
O	2.93870	0.85745	-0.64084
N	1.60611	1.05087	1.21370
O	0.46352	1.28364	1.77341
H	3.55795	1.00147	0.09110
H	1.03227	2.32781	-0.18048
H	-0.08834	2.40681	0.92537
O	0.13864	3.05983	0.12535
H	0.50355	3.87696	0.49282
**TS [6.2/6.3]w**			
-818.7069622	ImgFreq/IR inten: -1711.7737/149.2402		
O	-1.55031	1.58862	-0.32618
C	-0.51589	2.25439	0.28275
O	-0.34387	3.40888	0.02013
C	0.25164	1.49666	1.34413
H	-0.30897	1.64930	2.27439
H	1.21065	1.99747	1.47452
C	0.40406	-0.02295	1.12946
H	0.54826	-0.48052	2.11007
C	-0.85634	-0.59849	0.51629
C	-1.16147	-1.96026	0.61622
H	-0.46608	-2.61585	1.13214
C	-2.32992	-2.47907	0.06933
H	-2.54662	-3.53804	0.15732
C	-3.22241	-1.62796	-0.58512

H	-4.14011	-2.02014	-1.01051
C	-2.94129	-0.27120	-0.69173
H	-3.61711	0.41245	-1.19287
C	-1.76157	0.23248	-0.14499
C	1.66267	-0.43662	0.37246
O	2.50533	-1.18167	1.00490
N	1.94228	-0.09643	-0.86479
O	1.08016	0.84969	-1.44761
H	3.47864	-1.33604	0.22825
H	0.78689	0.45506	-2.28042
H	3.16646	-0.51171	-1.16774
O	4.08394	-1.18486	-0.80379
H	4.91311	-0.70410	-0.70410
**TS [6.2/7]$^{+}$w**			
-819.0805505	ImgFreq/IR inten: -164.8451/83.6549		
O	-0.54915	-1.09261	0.13240
C	-1.24055	-0.12272	0.77942
O	-2.48773	-0.42659	1.00717
C	-0.47822	0.64732	1.83680
H	-0.09265	-0.04521	2.58759
H	-1.14299	1.36581	2.31375
C	0.65632	1.30580	1.03219
H	1.23802	2.04202	1.58430
C	1.51139	0.18696	0.45112
C	2.89674	0.25362	0.30298
H	3.42661	1.14291	0.62819
C	3.59618	-0.81662	-0.24855
H	4.67369	-0.76099	-0.35004
C	2.91208	-1.95868	-0.66600
H	3.45651	-2.79180	-1.09544
C	1.52806	-2.04165	-0.53388
H	0.97993	-2.92094	-0.85134
C	0.84932	-0.96883	0.02257
C	-0.05796	1.90068	-0.15266
O	0.49542	2.80552	-0.91622
N	-1.19049	1.30788	-0.35300
O	-1.76824	1.49263	-1.59681
H	-0.04448	2.98320	-1.70746
H	-2.72628	1.53329	-1.44991
O	-3.66463	-2.21265	-0.52750
H	-2.84883	-1.14925	0.41683
H	-3.25017	-2.94789	-0.99255
H	-4.58148	-2.45189	-0.34904

**TS [7/8]$^+$w**				
-819.0097791	ImgFreq/IR inten: -1840.9797/1056.4662			
O		-0.52154	1.39577	-0.71800
C		0.65838	1.67540	0.34352
O		0.95958	2.81888	-0.14980
C		0.09893	1.29243	1.69782
H		-0.83302	1.80879	1.91870
H		0.83403	1.51569	2.47333
C		-0.07923	-0.22991	1.46393
H		-0.13298	-0.83102	2.37007
C		-1.30317	-0.44144	0.57109
C		-2.24073	-1.45062	0.78941
H		-2.08869	-2.15675	1.59918
C		-3.37769	-1.54176	-0.01169
H		-4.10456	-2.32357	0.17463
C		-3.58543	-0.62694	-1.04275
H		-4.47252	-0.69772	-1.66160
C		-2.65686	0.38362	-1.29437
H		-2.79600	1.09188	-2.10289
C		-1.53877	0.45311	-0.48394
C		1.17662	-0.56177	0.66673
O		1.64525	-1.74037	0.54088
N		1.61161	0.54699	0.11159
O		2.54083	0.59816	-0.89119
H		2.45033	-1.87402	-0.07498
H		2.69841	1.55530	-1.02112
H		-0.18799	2.53398	-0.87487
O		3.66270	-2.24535	-0.93414
H		4.07266	-1.66461	-1.58366
H		4.07208	-3.11634	-0.97852
**TS [7/8]$^+$2w**				
-819.0457766	ImgFreq/IR inten: -392.6944/79.8358			
O		0.36530	1.16578	-0.10171
C		1.56486	0.18478	0.59102
O		2.63578	0.81430	0.57014
C		0.82979	-0.43639	1.77256
H		0.38480	0.31984	2.41605
H		1.53924	-1.03252	2.35050
C		-0.22730	-1.31856	1.06351
H		-0.61779	-2.13372	1.67122
C		-1.32993	-0.44444	0.48424
C		-2.67339	-0.82390	0.47852
H		-2.96074	-1.77592	0.91352
C		-3.64112	0.01334	-0.06676
H		-4.68327	-0.28314	-0.05600

C	-3.26291	1.23432	-0.62582
H	-4.01185	1.88910	-1.05707
C	-1.92664	1.62462	-0.64112
H	-1.63084	2.56685	-1.09045
C	-0.96386	0.78695	-0.08353
C	0.55259	-1.83613	-0.12316
O	0.22547	-2.89280	-0.80726
N	1.51991	-1.01459	-0.38205
O	2.26922	-1.07937	-1.51738
H	0.77776	-3.02099	-1.59910
H	3.09376	-0.60279	-1.29803
H	2.50841	2.40621	0.12971
H	0.97212	2.41909	-0.25917
O	1.84158	3.06489	-0.24887
H	1.75771	3.86004	0.29705
**TS [8^{+}2w/8:H_3O^{+}]**			
-819.0948271	ImgFreq/IR inten: -565.7872/3670.1894		
O	-0.58889	-0.26831	1.67487
C	1.74675	-1.76937	-0.06437
O	2.57743	-2.50008	0.39216
C	0.37404	-1.97313	-0.64597
H	-0.25903	-2.44515	0.10617
H	0.43487	-2.65413	-1.49722
C	-0.13575	-0.55352	-1.04088
H	-0.16889	-0.46308	-2.13145
C	-1.49526	-0.16365	-0.49862
C	-2.57603	0.06144	-1.35249
H	-2.43303	-0.01821	-2.42587
C	-3.83302	0.38226	-0.84624
H	-4.66327	0.55052	-1.52184
C	-4.01342	0.48437	0.53098
H	-4.98776	0.73255	0.93673
C	-2.94541	0.27031	1.39999
H	-3.08738	0.35302	2.47408
C	-1.69463	-0.05023	0.88299
C	0.97830	0.37561	-0.59009
O	0.96132	1.62113	-0.67983
N	1.98944	-0.36407	-0.12788
O	3.15433	0.17124	0.36554
H	1.94193	2.34136	-0.25947
H	3.65266	-0.60919	0.68697
H	-0.81049	-0.18153	2.61029
O	2.82185	2.99373	0.14086
H	3.65066	2.52692	0.30803
H	2.96195	3.88924	-0.19189

Ethyl ester of 3-coumarin-carboxylic acid

**TS [2.1/2.2]**			
-1009.3959879	ImgFreq/IR inten: -2165.4786/564.3904		
O	-0.42107	0.59259	1.76819
C	-0.15167	-0.72609	1.50586
O	-0.18152	-1.51689	2.40225
C	0.18679	-1.06641	0.06061
H	0.68866	-2.03723	0.08643
C	-1.05275	-1.26946	-0.82409
C	1.11172	0.00610	-0.56112
H	1.19314	-0.21324	-1.63147
C	0.45371	1.35424	-0.36938
C	0.55575	2.39653	-1.29308
H	1.12367	2.23938	-2.20539
C	-0.06778	3.61979	-1.06222
H	0.01930	4.41859	-1.79051
C	-0.81033	3.80958	0.10423
H	-1.30043	4.75932	0.29018
C	-0.93173	2.78143	1.03337
H	-1.50358	2.90221	1.94621
C	-0.29774	1.56726	0.78713
C	2.48885	0.00142	0.07696
O	3.32780	-1.87752	-1.15614
N	3.35353	-1.08627	-0.25725
O	4.21098	-1.16000	0.74978
H	3.05898	0.92106	-0.06310
H	3.37219	-0.36356	1.23960
O	-0.98673	-1.39563	-2.02068
O	-2.18164	-1.31384	-0.11122
C	-3.41651	-1.54908	-0.84518
C	-4.54999	-1.54834	0.15652
H	-3.32322	-2.50293	-1.36792
H	-3.52159	-0.76276	-1.59515
H	-4.41663	-2.33194	0.90521
H	-5.49616	-1.72808	-0.36108
H	-4.62159	-0.58845	0.67244

**TS [2.1/2.2]w**			
-1085.8869486	ImgFreq/IR inten: -1383.0074/101.7790		
O	-0.42203	0.58579	1.70505
C	-0.23935	-0.73614	1.36795
O	-0.17390	-1.54862	2.24295
C	-0.09689	-1.03163	-0.11674
H	0.37003	-2.01608	-0.19412
C	-1.44409	-1.15364	-0.84257
C	0.79053	0.03159	-0.80485
H	0.74433	-0.16170	-1.88107

C	0.19344	1.38862	-0.50412
C	0.20452	2.45348	-1.40686
H	0.65211	2.30921	-2.38565
C	-0.35879	3.68214	-1.07074
H	-0.34450	4.49817	-1.78500
C	-0.95014	3.85501	0.18148
H	-1.39467	4.80804	0.44795
C	-0.97946	2.80446	1.09375
H	-1.43472	2.91098	2.07180
C	-0.40702	1.58635	0.74029
C	2.23181	-0.01107	-0.32654
O	2.56637	-1.99594	-1.44658
N	3.01750	-1.06761	-0.79276
O	4.26854	-1.08684	-0.41006
H	2.78517	0.92174	-0.40496
H	2.75985	-0.17251	1.22208
O	-1.55177	-1.13969	-2.04221
O	-2.46228	-1.30882	0.01223
C	-3.78355	-1.49508	-0.56648
C	-4.76244	-1.66286	0.57484
H	-3.74946	-2.37109	-1.21710
H	-4.01080	-0.62514	-1.18588
H	-5.76961	-1.81028	0.17552
H	-4.77696	-0.77963	1.21713
H	-4.50850	-2.52958	1.18858
H	4.19916	-0.73043	0.92429
O	3.64422	-0.43116	1.83634
H	4.01142	0.35637	2.25743
**TS [2.2$^+$2w/3.1$^+$]** -1086.2166867	ImgFreq/IR inten: -1423.7935/1170.4711		
O	-1.02935	-1.47900	1.70118
C	0.21885	-0.95961	1.81259
O	0.90205	-1.24335	2.74835
C	0.70014	0.04697	0.76247
C	1.94231	-0.39556	-0.01995
H	1.09104	0.85976	1.38479
C	-0.36576	0.61524	-0.23255
H	0.07365	0.69310	-1.22961
C	-1.60530	-0.24874	-0.32985
C	-2.49642	-0.11929	-1.40615
H	-2.28795	0.60590	-2.18642
C	-3.62453	-0.92500	-1.50121
H	-4.29902	-0.81416	-2.34235
C	-3.87062	-1.89069	-0.52266
H	-4.74498	-2.52765	-0.59257

C	-2.98516	-2.05524	0.53455
H	-3.13941	-2.81087	1.29575
C	-1.86137	-1.23765	0.61817
C	-0.66486	2.03199	0.21703
N	0.39426	3.08719	-0.03732
O	1.30890	2.81340	-1.02466
O	1.00880	3.38051	1.19393
H	-0.98753	2.13078	1.25158
H	1.86542	2.01421	-0.78551
O	-1.62608	2.76447	-0.66048
H	-2.52392	2.88809	-0.30867
H	-0.82157	3.61732	-0.61217
H	1.47856	4.22095	1.05697
O	2.56443	0.43805	-0.66264
O	2.22417	-1.66438	0.07067
C	3.43700	-2.18262	-0.59839
H	3.74320	-2.99760	0.05396
H	4.18761	-1.39446	-0.58117
C	3.11447	-2.65235	-2.00030
H	4.00909	-3.10345	-2.43736
H	2.81210	-1.82467	-2.64487
H	2.32806	-3.40965	-1.99442
**TS [2.2⁺w/3.2⁺]**			
-1086.2448778	ImgFreq/IR inten: -820.6690/2136.9112		
O	-0.42009	0.16650	1.78406
C	0.29870	-0.78281	1.26769
O	1.22023	-1.29170	1.96435
C	0.23783	-1.02623	-0.20531
C	-1.14990	-1.37231	-0.78435
H	0.87581	-1.87135	-0.45885
C	0.80635	0.29667	-0.76530
H	0.87067	0.22345	-1.85134
C	-0.18939	1.36460	-0.33448
C	-0.55722	2.45995	-1.11339
H	-0.08661	2.60701	-2.07967
C	-1.54213	3.34529	-0.67210
H	-1.81845	4.18982	-1.29266
C	-2.18166	3.14134	0.54897
H	-2.94966	3.82837	0.88455
C	-1.83747	2.05283	1.35122
H	-2.30558	1.87764	2.31252
C	-0.85714	1.19843	0.88760
C	2.23713	0.57909	-0.20331
N	2.94768	-0.70762	0.19969
O	4.20356	-0.40195	0.70880
O	3.09161	-1.48013	-0.98094

H	2.17772	1.11030	0.75055
H	4.66897	0.08910	0.00179
O	3.02739	1.27073	-1.11542
H	3.25521	2.14954	-0.78879
H	2.11500	-1.30005	1.26417
H	3.53137	-2.29950	-0.69783
O	-1.43624	-1.15388	-1.92789
O	-1.88127	-2.00299	0.12858
C	-3.19588	-2.53146	-0.28308
H	-3.34545	-3.37303	0.39062
H	-3.10117	-2.89457	-1.30557
C	-4.27913	-1.48325	-0.14230
H	-5.24406	-1.93431	-0.38819
H	-4.12075	-0.64615	-0.82433
H	-4.33891	-1.10828	0.88170

TS [3.1$^+$/3.2$^+$]
-1086.2410391 ImgFreq/IR inten: -1144.7969/2716.0642

O	-0.75858	0.20111	1.85642
C	-0.13567	-0.88543	1.48553
O	0.54777	-1.51476	2.31787
C	-0.00186	-1.17710	0.01752
C	-1.31743	-1.30236	-0.77931
H	0.50326	-2.13655	-0.10214
C	0.86583	-0.00483	-0.48378
H	0.99958	-0.12225	-1.55883
C	0.06858	1.25801	-0.18529
C	0.07398	2.38302	-1.00904
H	0.72153	2.40149	-1.87927
C	-0.75993	3.46665	-0.73522
H	-0.74414	4.33256	-1.38674
C	-1.62186	3.43395	0.35964
H	-2.27405	4.27458	0.56605
C	-1.65101	2.32187	1.20043
H	-2.29821	2.27617	2.06818
C	-0.80835	1.26642	0.90762
C	2.27032	-0.01313	0.18969
N	3.32132	0.19854	-0.75012
O	3.31078	-0.72795	-1.79633
O	4.52494	0.06146	-0.01394
H	2.37477	0.73925	0.96842
H	3.53697	-1.60510	-1.43245
H	5.15648	0.66931	-0.42699
O	2.50974	-1.36178	0.90911
H	3.38726	-1.32814	1.32795
H	1.62903	-1.63744	1.68815

O	-1.37527	-1.07759	-1.95633
O	-2.28336	-1.76342	0.00594
C	-3.59102	-2.06162	-0.60815
H	-3.99125	-2.84618	0.03117
H	-3.40605	-2.46081	-1.60449
C	-4.47604	-0.83360	-0.63448
H	-5.45664	-1.11006	-1.03071
H	-4.06480	-0.05498	-1.27927
H	-4.62430	-0.42927	0.36912
**TS [2.2/4']**			
-1009.3845499	ImgFreq/IR inten: -774.9873/21.7264		
O	-1.18943	-2.29431	-0.24983
C	0.09088	-2.11347	0.17343
O	0.87363	-3.01367	0.09284
C	0.41016	-0.75205	0.79101
H	0.00767	-0.77917	1.80995
C	1.92320	-0.58410	0.93097
C	-0.28183	0.40602	0.01047
H	0.18228	0.51059	-0.97222
C	-1.76164	0.07290	-0.12385
C	-2.76576	1.04570	-0.17483
H	-2.48585	2.09361	-0.10717
C	-4.10030	0.67321	-0.31347
H	-4.86833	1.43762	-0.35264
C	-4.44552	-0.67493	-0.40310
H	-5.48433	-0.96783	-0.51095
C	-3.45814	-1.65437	-0.36112
H	-3.69469	-2.70912	-0.44005
C	-2.13008	-1.26904	-0.22331
C	-0.10399	1.67134	0.78109
N	0.46270	2.79085	0.35313
O	1.00197	2.69072	-0.95071
O	-0.73573	3.56609	0.44742
H	1.50628	3.51102	-1.04834
H	-0.43303	1.72062	1.81217
O	2.49016	-0.54793	1.99335
O	2.50540	-0.46075	-0.26304
C	3.95482	-0.33885	-0.27780
H	4.37120	-1.21424	0.22378
H	4.22870	0.54564	0.30077
C	4.39039	-0.24166	-1.72307
H	5.47862	-0.14855	-1.77252
H	3.95149	0.63161	-2.21071
H	4.09991	-1.13311	-2.28286

**TS [2.2/4'']**			
-1009.3866489	ImgFreq/IR inten: -897.0411/42.6806		
O	0.12374	0.46193	1.71061
C	-0.32071	-0.77258	1.27250
O	-0.67531	-1.57055	2.08358
C	-0.31564	-0.99247	-0.23453
H	-0.24140	-2.07485	-0.37962
C	-1.61565	-0.53837	-0.91319
C	0.87775	-0.32134	-0.92439
H	0.70271	-0.33872	-2.00576
C	0.99118	1.12512	-0.46264
C	1.46726	2.15706	-1.27570
H	1.76770	1.93378	-2.29515
C	1.54118	3.46108	-0.79827
H	1.90743	4.25378	-1.44098
C	1.13175	3.74444	0.50661
H	1.18457	4.75973	0.88477
C	0.64722	2.73255	1.32759
H	0.31885	2.92870	2.34170
C	0.58539	1.42954	0.83885
C	2.20301	-0.95754	-0.66948
N	2.36864	-2.00054	0.12613
O	3.73346	-2.23184	0.41881
O	1.76925	-2.95827	-0.73874
H	3.71173	-2.77134	1.22229
H	3.09054	-0.47134	-1.08102
O	-1.68449	-0.26350	-2.08595
O	-2.64529	-0.52909	-0.06620
C	-3.95142	-0.19790	-0.61650
H	-4.16938	-0.90278	-1.42102
H	-3.89491	0.80230	-1.05066
C	-4.95465	-0.28264	0.51255
H	-5.95221	-0.04215	0.13502
H	-4.71324	0.42263	1.31065
H	-4.98386	-1.28707	0.93955
**TS [6.1/6.2]**			
-1009.3803317	ImgFreq/IR inten: -2358.3760/451.6270		
O	-0.06854	0.43936	1.68772
C	-0.41342	-0.85204	1.38164
O	-0.74195	-1.59036	2.26275
C	-0.34948	-1.24175	-0.08829
C	-1.64319	-0.92246	-0.84874
H	-0.24827	-2.32896	-0.11370
C	0.85550	-0.60347	-0.82359
H	0.67244	-0.74156	-1.89308
C	0.90334	0.87263	-0.50323

C	1.40390	1.82778	-1.39334
H	1.77491	1.50101	-2.36017
C	1.42830	3.17725	-1.05907
H	1.82015	3.90380	-1.76250
C	0.94224	3.59163	0.18325
H	0.95737	4.64257	0.45191
C	0.43343	2.66130	1.08115
H	0.05082	2.95494	2.05178
C	0.41960	1.31301	0.73057
C	2.18141	-1.29023	-0.52479
O	2.06858	-2.71079	-0.56152
N	2.81917	-0.90929	0.68186
O	4.04887	-1.10431	0.46230
H	2.50184	-3.05935	-1.34674
H	3.71933	-1.28859	-0.76648
O	-1.75442	-1.08336	-2.03937
O	-2.61220	-0.47145	-0.04795
C	-3.89610	-0.17664	-0.66501
H	-4.26538	-1.09117	-1.13315
H	-3.73486	0.56314	-1.45156
C	-4.81934	0.32731	0.42239
H	-5.79940	0.55538	-0.00542
H	-4.42855	1.23747	0.88231
H	-4.95435	-0.42234	1.20475
**TS [6.2/6.3]**			
-1009.4706740	ImgFreq/IR inten: -1882.8793/388.3928		
O	-0.04427	0.29626	1.74619
C	-0.30341	-0.95570	1.26302
O	-0.56556	-1.83733	2.03035
C	-0.19444	-1.12764	-0.24575
C	-1.45031	-0.67914	-1.00602
H	-0.10378	-2.20011	-0.41966
C	1.03357	-0.38098	-0.80309
H	0.96782	-0.40479	-1.89573
C	0.96566	1.06020	-0.33394
C	1.42352	2.13817	-1.09241
H	1.87598	1.94810	-2.05977
C	1.31141	3.44036	-0.61587
H	1.67420	4.26814	-1.21507
C	0.73250	3.67708	0.63193
H	0.64234	4.69059	1.00799
C	0.26630	2.61619	1.40105
H	-0.18339	2.77059	2.37508
C	0.39039	1.32084	0.90956
C	2.36007	-1.00300	-0.44631
O	3.48247	-0.39776	-0.63062

N	2.68478	-2.16747	0.07744
O	1.80882	-3.26024	0.14928
H	3.87375	-1.59225	-0.16488
H	1.77208	-3.50534	1.08586
O	-1.46999	-0.52266	-2.20107
O	-2.50183	-0.53356	-0.19688
C	-3.76878	-0.17358	-0.81783
H	-4.03172	-0.95854	-1.52956
H	-3.62290	0.75311	-1.37582
C	-4.79252	-0.02979	0.28651
H	-5.76159	0.23513	-0.14486
H	-4.50724	0.75510	0.99011
H	-4.90972	-0.96312	0.84102

TS [6.1/6.2]w

-1085.8894675 ImgFreq/IR inten: -1591.9468/751.6748

O	-0.27101	0.20815	1.78077
C	-0.69254	-1.01883	1.33899
O	-1.09611	-1.81964	2.13022
C	-0.57786	-1.26624	-0.15893
C	-1.77683	-0.74951	-0.96300
H	-0.56897	-2.34902	-0.29058
C	0.73258	-0.66302	-0.73172
H	0.67039	-0.75294	-1.81816
C	0.75444	0.79592	-0.34169
C	1.18873	1.81571	-1.19175
H	1.57814	1.55669	-2.17061
C	1.13698	3.14865	-0.79519
H	1.48479	3.92662	-1.46624
C	0.62748	3.48006	0.46122
H	0.58565	4.51698	0.77744
C	0.15411	2.48370	1.30822
H	-0.26008	2.71248	2.28330
C	0.21331	1.15777	0.89392
C	1.92376	-1.53849	-0.25601
O	1.66393	-2.87166	-0.55637
N	2.68769	-1.36692	0.83592
O	3.09798	-0.17379	1.11440
H	2.20499	-3.41614	0.03587
H	3.02713	-0.89176	-1.06058
H	3.58101	0.33022	-0.23184
O	3.74171	0.04715	-1.23589
H	4.65591	-0.25590	-1.32731
O	-1.78679	-0.70227	-2.16793
O	-2.79972	-0.39882	-0.17791
C	-4.01476	0.04980	-0.83973
H	-4.35050	-0.74484	-1.50887

H	-3.76905	0.92166	-1.44926
C	-5.03065	0.36457	0.23620
H	-5.96236	0.70125	-0.22660
H	-4.67380	1.15651	0.89815
H	-5.24955	-0.51692	0.84233
**TS [6.2/6.3]w**			
-1085.9655039	ImgFreq/IR inten: -1717.4712/137.0958		
O	-0.61659	0.68170	1.74190
C	-0.51235	-0.67707	1.60938
O	-0.65734	-1.37194	2.57035
C	-0.28650	-1.20553	0.19804
C	-1.62110	-1.39657	-0.55656
H	0.11610	-2.21489	0.31667
C	0.65933	-0.34555	-0.67394
H	0.42496	-0.59128	-1.71061
C	0.37587	1.12462	-0.44512
C	0.71167	2.08984	-1.40047
H	1.20738	1.77242	-2.31300
C	0.41769	3.43411	-1.19964
H	0.68772	4.16639	-1.95261
C	-0.23425	3.83168	-0.03072
H	-0.47426	4.87695	0.13291
C	-0.58295	2.88881	0.92886
H	-1.08883	3.16941	1.84571
C	-0.27246	1.54641	0.71688
C	2.13871	-0.70278	-0.55949
O	2.70051	-1.18898	-1.61302
N	2.87538	-0.54246	0.51659
O	2.18091	-0.18466	1.68508
H	3.87443	-1.47578	-1.27767
H	2.62463	0.60684	2.01981
H	4.07106	-1.07996	0.31145
O	4.80309	-1.54519	-0.51103
H	5.09003	-2.44579	-0.32377
O	-1.67276	-1.65085	-1.73354
O	-2.67672	-1.28244	0.25166
C	-3.99762	-1.49826	-0.32309
H	-4.59398	-1.83495	0.52424
H	-3.92642	-2.30044	-1.05766
C	-4.55031	-0.22274	-0.93120
H	-5.57140	-0.39859	-1.28205
H	-3.95231	0.10148	-1.78457
H	-4.58002	0.58254	-0.19378
**TS [6.2/7]$^{+}$w**			
-1086.3392486	ImgFreq/IR inten: -156.1182/85.2664		
O	-0.03895	0.61962	1.01697

C	0.08204	1.21589	-0.19435
O	0.78255	2.31514	-0.17355
C	0.22495	0.28862	-1.38262
C	1.44070	-0.66226	-1.36529
H	0.35559	0.87696	-2.28955
C	-1.09055	-0.50942	-1.35670
H	-1.26239	-1.13239	-2.23281
C	-1.12028	-1.29639	-0.05272
C	-1.66032	-2.57548	0.08031
H	-2.07992	-3.07127	-0.78891
C	-1.65224	-3.21604	1.31659
H	-2.06406	-4.21417	1.40893
C	-1.11225	-2.57594	2.43256
H	-1.10598	-3.07444	3.39499
C	-0.57520	-1.29562	2.32226
H	-0.15042	-0.78329	3.17743
C	-0.58685	-0.67592	1.08233
C	-2.14059	0.55690	-1.18791
O	-3.40562	0.33137	-1.42602
N	-1.62990	1.62893	-0.67406
O	-2.52376	2.53486	-0.13073
H	-3.96109	1.07937	-1.14114
H	-2.16699	3.41722	-0.31875
O	0.98496	3.65438	2.08389
H	0.85549	2.73868	0.73009
H	0.94332	3.30070	2.97929
H	1.51759	4.45788	2.10538
O	1.44703	-1.66450	-2.03015
O	2.43361	-0.20890	-0.61217
C	3.67240	-0.99818	-0.60942
H	4.01838	-1.07314	-1.64100
H	3.42650	-2.00099	-0.25767
C	4.66298	-0.29212	0.28629
H	5.59822	-0.85693	0.30584
H	4.29326	-0.21935	1.31138
H	4.88454	0.71269	-0.07898
**TS [7/8]$^+$w**			
-1086.2689347	ImgFreq/IR inten: -1839.7895/970.5662		
O	0.08406	-0.18210	-1.35989
C	-0.28746	-1.37564	-0.37478
O	0.07164	-2.30311	-1.18120
C	0.22071	-0.98673	1.01350
C	1.70454	-0.69769	1.24461
H	-0.05454	-1.78843	1.69948
C	-0.69001	0.25114	1.25670
H	-0.77698	0.53895	2.30229

C	-0.19270	1.40713	0.38806
C	-0.11509	2.72247	0.84643
H	-0.44738	2.95874	1.85166
C	0.40878	3.72345	0.02974
H	0.47286	4.73992	0.40083
C	0.85841	3.41860	-1.25372
H	1.26763	4.20001	-1.88631
C	0.78350	2.11384	-1.74295
H	1.11288	1.86521	-2.74687
C	0.26119	1.14065	-0.91179
C	-2.01762	-0.24462	0.70225
O	-3.14873	0.26095	1.01149
N	-1.76152	-1.20566	-0.15642
O	-2.66400	-1.72984	-1.03926
H	-3.97845	-0.10447	0.54178
H	-2.16188	-2.44549	-1.48395
H	0.54867	-1.17193	-1.84882
O	-5.32543	-0.56003	-0.03372
H	-5.43594	-1.19739	-0.74801
H	-6.18968	-0.23300	0.24249
O	2.07253	-0.17786	2.26541
O	2.47453	-1.10819	0.24462
C	3.92581	-0.95481	0.42198
H	4.20092	-1.47367	1.34082
H	4.13295	0.10801	0.55418
C	4.60070	-1.53612	-0.79811
H	5.68416	-1.44880	-0.68617
H	4.31530	-1.00308	-1.70785
H	4.35886	-2.59345	-0.92156
**TS [7/8]$^+$2w**			
-1086.3192152	ImgFreq/IR inten: -241.5133/103.9918		
O	1.12946	-0.32845	1.23109
C	1.04494	-1.39068	0.09142
O	1.32662	-2.52020	0.52634
C	-0.18667	-1.01586	-0.77318
C	-1.53165	-1.02251	-0.06197
H	-0.25155	-1.76060	-1.57295
C	0.24861	0.35502	-1.36575
H	-0.31872	0.67145	-2.23613
C	0.27109	1.39336	-0.25631
C	-0.10878	2.72419	-0.44094
H	-0.45332	3.04948	-1.41684
C	-0.05195	3.62649	0.61636
H	-0.35786	4.65561	0.46734
C	0.39782	3.20050	1.86537
H	0.44650	3.90037	2.69119

C	0.79580	1.88177	2.06552
H	1.16621	1.54967	3.02949
C	0.72880	0.98223	1.00505
C	1.69573	0.07247	-1.71632
O	2.34889	0.74196	-2.62840
N	2.14074	-0.84886	-0.93741
O	3.42832	-1.26955	-0.88320
H	3.29221	0.50633	-2.67057
H	3.37496	-2.11070	-0.38158
H	0.53154	-2.80744	2.42565
H	0.60697	-1.26384	2.51646
O	0.04590	-2.05032	2.80338
H	-0.77994	-1.92991	2.20961
O	-1.74127	-1.45660	1.06538
O	-2.47668	-0.54769	-0.83575
C	-3.86303	-0.53566	-0.32463
H	-3.86647	0.06146	0.58754
H	-4.12253	-1.56351	-0.07029
C	-4.74169	0.04068	-1.40812
H	-5.77578	0.05853	-1.05556
H	-4.70723	-0.56446	-2.31592
H	-4.45414	1.06438	-1.65529

**TS [8^+2w/8:H_3O^+]**			
-1086.3464809	ImgFreq/IR inten: -104.2035/161.0798		
O	0.84579	0.90551	1.45784
C	1.09635	-1.93547	0.02528
O	1.28568	-2.90840	0.70111
C	-0.12253	-1.32698	-0.62093
C	-1.34129	-1.30988	0.31559
H	-0.37555	-1.96054	-1.47510
C	0.36048	0.08689	-1.14785
H	0.22617	0.12211	-2.23224
C	-0.34045	1.29120	-0.54810
C	-1.27778	2.02020	-1.28600
H	-1.47004	1.75683	-2.32216
C	-1.98545	3.06456	-0.69726
H	-2.71679	3.61806	-1.27582
C	-1.74970	3.39308	0.63755
H	-2.29492	4.20712	1.10234
C	-0.80710	2.68763	1.38199
H	-0.60850	2.95155	2.41636
C	-0.11116	1.64000	0.78741
C	1.86881	0.08940	-0.88648
O	2.67072	0.99953	-1.12165
N	2.18315	-1.11810	-0.35624
O	3.43940	-1.46087	0.11094

H	3.93901	1.00447	-0.53374
H	3.27435	-2.27699	0.63278
H	0.58282	0.75597	2.37450
O	4.80041	0.75920	0.09365
H	4.59312	-0.16152	0.38487
H	5.67203	0.80771	-0.32973
O	-2.45079	-1.45929	-0.39987
O	-1.27683	-1.17520	1.50946
C	-3.72170	-1.43995	0.33299
C	-4.83078	-1.66448	-0.66834
H	-3.79861	-0.47491	0.83613
H	-3.67691	-2.22109	1.09285
H	-5.79330	-1.64531	-0.15130
H	-4.73301	-2.63382	-1.16111
H	-4.84311	-0.88402	-1.43189
